# Body size and trophic structure explain global asymmetric response of tetrapod diversity to climate effects

**DOI:** 10.1002/ece3.11047

**Published:** 2024-02-20

**Authors:** Reginaldo A. F. Gusmão, Geiziane Tessarolo, Ricardo Dobrovolski, Thiago Gonçalves‐Souza

**Affiliations:** ^1^ Graduate Program in Ethnobiology and Nature Conservation, Department of Biology Federal Rural University of Pernambuco Recife Brazil; ^2^ Laboratory of Biogeography and Aquatic Ecology State University of Goiás Anápolis Brazil; ^3^ Biology Institute Federal University of Bahia Salvador BA Brazil; ^4^ Institute for Global Change Biology, School for Environment and Sustainability University of Michigan Ann Arbor Michigan USA; ^5^ Department of Ecology and Evolutionary Biology University of Michigan Ann Arbor Michigan USA

**Keywords:** climate instability, contemporary climate, food web theory, functional traits, macroecology, metabolic theory, species pattern

## Abstract

Although climate‐based hypotheses are widely used to explain large‐scale diversity patterns, they fall short of explaining the spatial variation among taxonomic groups. Integrating food web and metabolic theories into macroecology is a promising step forward, as they allow including explicit taxon‐specific traits that can potentially mediate the relationship between climate and diversity. Our investigation focuses on the role of body size and trophic structure in mediating the influence of contemporary climate and historical climate change on global tetrapods species richness. We used piecewise structural equation modeling to assess the direct effects of contemporary climate and climate instability of species richness and the indirect effects of climate on tetrapod richness mediated by community‐wide species traits. We found that birds and mammals are less sensitive to the direct effect of contemporary climate than amphibians and squamates. Contemporary climate and climate instability favored the species richness of mammals and amphibians. However, for birds and squamates, this link is only associated with contemporary climate. Moreover, we showed that community‐wide traits are correlated with species richness gradients. However, we highlight that this relationship is dependent upon the specific traits and taxonomic groups. Specifically, bird communities with smaller bodies and bottom‐heavy structures support higher species richness. Squamates also tend to be more diverse in communities with prevalence of smaller bodies, while mammals are correlated with top‐heavy structures. Moreover, we showed that higher contemporary climate and climate instability reduce the species richness of birds and mammals through community‐wide traits and indirectly increase squamate species richness. We also showed that body size and trophic structure are driving a global asymmetric response of tetrapod diversity to climate effects, which highlights the limitation to use the “typical” climate‐based hypotheses. Furthermore, by combining multiple theories, our research contributes to a more realistic and mechanistic understanding of diversity patterns across taxonomic groups.

## Graphical abstract

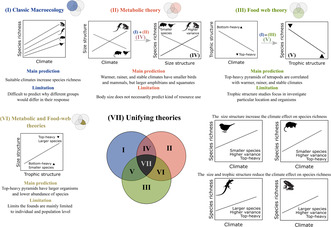

## 1 INTRODUCTION

The most difficult challenge for ecological theory has been explaining the nonhomogeneous distribution of species, which has encouraged the development of various hypotheses with varying degrees of support (Pianka, 1996; Willig et al., 2003). Climate‐based hypotheses have been shown to be the most effective in explaining patterns of global diversity (Gillman & Wright, 2014). However, empirical studies criticized these hypotheses because species from different trophic levels and metabolic rates respond asymmetrically to climate (Brown et al., 2004; Voigt et al., 2003). Thus, a tangle of diffuse explanations requires synthesis to understand the mechanisms underlying the climate‐based hypotheses (McGill, 2019).

**FIGURE 1:**
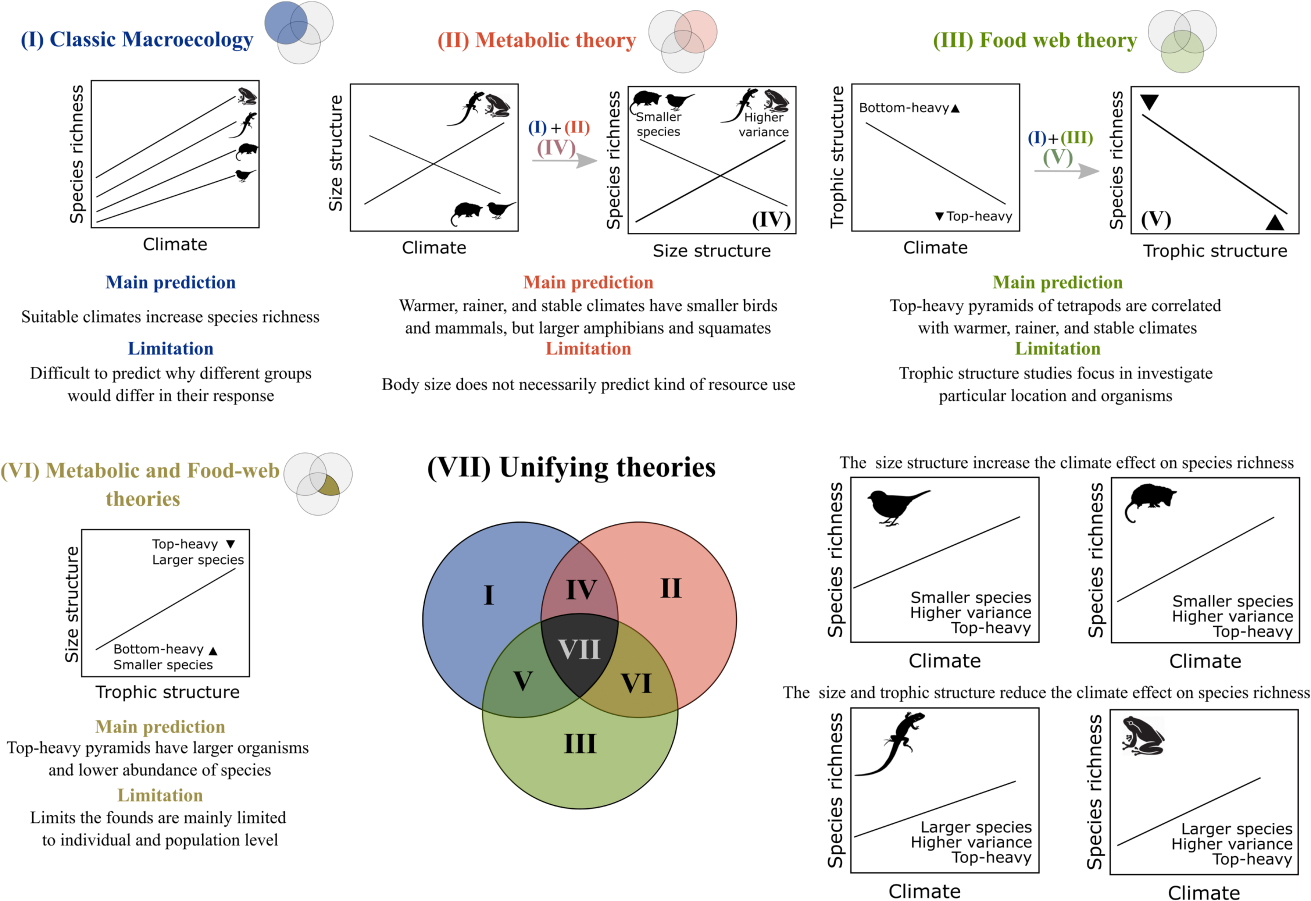
Conceptual framework containing predictions to each separate theory and predictions integrating the theories to the asymmetric response by tetrapods to climate.

Contemporary temperature and precipitation (hereafter, contemporary climate) have been widely established in the literature as the main drivers of species richness (Evans et al., 2005; Whittaker et al., 2007; Table 1a). Moreover, climate change from the Last Glacial Maximum (LGM, a measure of climate instability) also explains broad scale diversity patterns, adding fuel to a debate concerning whether contemporary climate or climate instability are better predictors of global species richness (Araújo & Rahbek, 2006; Santos et al., 2020; Table 1b). Despite this central relevance of climate‐based hypotheses to macroecology, this theory has yet not fully untangled the strength and direction of the asymmetric effect of contemporary climate and climate instability on species richness, which can vary depending on species traits and taxonomic groups (Barreto et al., 2021; Ficetola et al., 2021; Figure 1 (I)). For instance, bird and mammal diversity are less sensitive to the contemporary climate because they have a broader thermal tolerance range than amphibians and squamates (Buckley et al., 2012; Ficetola et al., 2021). However, compared to birds and mammals, amphibians and squamates are more vulnerable to climate instability due to their smaller bodies reducing their ability to disperse (Ficetola et al., 2021). Additionally, the responses to climate could also vary within the same taxon when comparing across trophic levels (Sandel et al., 2011; Voigt et al., 2003). For example, species of higher trophic levels are more sensitive to climate than lower trophic levels mainly because higher trophic levels require more energy and resources (Voigt et al., 2003). These trait or taxon‐dependent responses to climate suggest that two major questions remain unanswered: How do the mechanisms involved in the relationship between climate and species richness differ between taxonomic groups? How do community‐wide species traits (e.g., size and trophic structure at the community scale) explain the disparities in global diversity patterns among tetrapods?

**TABLE 1:** The assumptions and predictions of effect of theoretical variables and its respective predictor variables on tetrapod species richness.

Theoretical variable	Predictor variable	Premises	Predictions
(a) Contemporary climate	Temperature	Temperature is positively linked to increased energy availability, favoring diversification and the number of coexisting species (Clarke & Gaston, 2006; Gillman & Wright, 2014; Figure 1 (I) Classic macroecology). Furthermore, increasing temperature decreases the average size of species because smaller bodies have a reduced heat loss (Phillips & Heath, 1995). The temperature tends to reduce the variance of body size favoring an evolutionary convergence among coexisting species to increase the survival rate (Araújo et al., 2013; Rapacciuolo et al., 2017; Figure 1 (II) Metabolic theory). Lastly, warmer climates have higher energy availability allowing more species of higher trophic levels favoring top‐heavy pyramids (Danet et al., 2021; Figure 1 (III) Food web theory)	Higher temperature favors tetrapod species richness and positive indirect effects on species richness by reducing body size and size variation. Moreover, temperatures reduce species richness indirectly by decreasing size variation and increasing the presence of higher trophic levels (see community‐wide species traits)
	Precipitation	Precipitation is linked to increased numbers of individuals, which increase diversification rate, and ultimately favoring species diversity (Gillman et al., 2015; Tieleman et al., 2003; Figure 1 (I) Classic macroecology). Wetter climates have more resource availability favoring larger species, and higher body size variance which, in turn, increases the number of species at each trophic level (bottom‐heavy pyramids) due to greater niche availability (Hopwood et al., 2016; Figure 1 (II) Metabolic theory and (III) Food web theory)	Precipitation may have a direct and positive effect on tetrapod species richness and may also have a negative indirect effect through body size and a positive indirect effect by size variation and trophic structure (see community‐wide species traits)
(b) Climate instability	Temperature anomaly	Higher temperature anomaly may be associated with a less diverse community due to a lower speciation rate and higher extinction rate (Hortal et al., 2011; Figure 1 (I) Classic macroecology). These communities have larger species that are highly resistant to climatic anomaly, which favors convergence of body size (Buckley et al., 2012; Hopwood et al., 2016; Figure 1 (II) Metabolic theory). On the other hand, food‐web theory shows that predators are more sensitive to climatic anomalies, so higher temperature anomalies can decline the number of species of higher trophic level (Voigt et al., 2003; Figure 1 (III) Food web theory)	Temperature anomalies decrease tetrapod species richness. Higher temperature anomalies reduce species richness indirectly through body size and variation in body size. Conversely, temperature anomalies indirectly increase species richness by decreasing the presence of species from higher trophic levels
	Precipitation anomaly	Reduced precipitation anomaly is linked to lower productivity and higher water stress, which leads to a reduction in the number of individuals and species (Araújo et al., 2008; Figure 1 (I) Classic macroecology). There is an evolutionary trend toward larger species that support conditions with water stress and there is reduction of body size variance (Peralta‐Maraver & Rezende, 2021; Tieleman et al., 2003; Figure 1 (II) Metabolic theory). Another effect of lower precipitation anomaly is that the reduction in resources reduces predator species through a bottom‐up effect (Clarke & Gaston, 2006; Voigt et al., 2003; Figure 1 (III) Food web theory)	Higher precipitation anomalies reduce tetrapod species richness. Precipitation anomalies reduce species richness indirectly by favoring larger species and reducing body size variance. Precipitation anomalies, on the other hand, increase species richness by reducing higher trophic level species
(c) Community‐wide species traits	Body size	Regions dominated with larger species have a smaller number of individuals per species which, in turn, decreases species richness (Evans et al., 2005). Furthermore, due to higher species requiring more energy, the energy available to support a greater number of trophic levels is reduced (Brown et al., 2004; Evans et al., 2005)	Larger body size directly reduces tetrapod species richness. Moreover, regions dominated with larger species have fewer species richness of predators and omnivores
	Body size variance	Body size variance reduces interspecific competition due possibly best division of the niche, resulting in greater species coexistence (Evans et al., 2005; Figure 1 (I) + (III)). This lower competition allows higher species richness and trophic levels	Body size variance may directly increase tetrapod species richness. Moreover, higher body size variance favors bottom‐heavy pyramids
	Trophic structure	The energy demand is proportional to the trophic level; therefore, communities with more species at higher trophic levels require more energy and have greater top‐down control (Evans et al., 2005). However, bottom‐heavy chains tend to have more species richness than top‐heavy chains due stronger effect of bottom‐up control on species richness (Danet et al., 2021; Sandom et al., 2013; Figure 1 (I) + (III))	Regions with top‐heavy pyramids have lower tetrapod species richness

The number of species supported by a community can be determined by the link between climate and size structure (i.e., body size and variance of body size) of a community (Hillebrand & Azovsky, 2001). Communities dominated by larger species tend to have lower species richness because larger species may have lower generational rate and population size resulting in a reduced speciation rate and increased extinction rate (Brown et al., 2004; Evans et al., 2005; Peralta‐Maraver & Rezende, 2021). Metabolic theory allows for more refined predictions regarding the relationship between climate and species richness within each tetrapod group. For instance, the theory predicts regions characterized by lower temperatures, drier and higher climatic instability are likely to host larger‐bodied bird and mammal species (Brown et al., 2004; Phillips & Heath, 1995). This increase in body size is attributed to the organisms' reduced surface‐to‐volume ratio, which reduces heat and water losses improving survival and reproductive success, in accordance with Bergmann's rule (Phillips & Heath, 1995; Rapacciuolo et al., 2017). Bergmann's rule expresses that species tend to be larger in higher latitudes (closer to the poles) and smaller in lower latitudes (closer to the equator), mainly to mammals and birds (Rapacciuolo et al., 2017). In contrast, there is a reverse latitudinal pattern of body size distribution to amphibians and squamates because larger‐bodied species in higher temperatures have greater potential for heat gain (Ashton & Feldman, 2003; Olalla‐Tarraga et al., 2006; Rapacciuolo et al., 2017; see Table 1c). As a result, the conclusions of Bergmann's rule tend to be either ambiguous or limited, particularly in the case of some taxonomic groups such as ectotherms. This ambiguity arises due to the existence of either positive, negative, or nonlinear patterns in body size‐latitude correlation, or sometimes no discernible pattern at all (Ashton & Feldman, 2003; Johnson et al., 2023; Meiri & Dayan, 2003; Rapacciuolo et al., 2017). This lack of generality may be caused by the fact that the body size is determined by multiple simultaneous pressures, which are linked with other traits such as body size variation and trophic level. Therefore, these distinct relationships between climate and body size within tetrapod groups might determine the indirect effect of climate on species richness through body size and the relationship between multiple traits (see Figure 1 (II)).

Additionally, the climate also might determine the species richness of a community through the body size variance in a community. For instance, studies suggest that colder and drier regions favor an evolutionary convergence to larger‐bodied species due to climatic pressure acting as a stronger filter (Bothwell et al., 2015; Rapacciuolo et al., 2017; Read et al., 2018; Table 1c). As a result, this convergence leads to increased niche overlap and competition, particularly among larger species that have higher energetic demands for food resources (Hopwood et al., 2016; Pawar, 2015; Read et al., 2018). Hence, the intensified competition driven by climate limits the coexistence of species, ultimately resulting in a reduction in species richness (Hopwood et al., 2016; Pawar, 2015; see Figure 1 (IV)).

In addition to altering the size structure, climate also has a significant impact on the trophic structure, which in turn can affect the number of species in a region. Based on food‐web theory, precipitation also favors plant biomass, which might cascade up throughout the trophic chain, allowing the community to support a greater number of species at each trophic level (Hopwood et al., 2016). As a result, precipitation can result in more bottom‐heavy food‐web pyramids (i.e., communities with higher biomass or species richness on lower trophic levels) with greater bottom‐up control (Hatton et al., 2015). Higher temperatures, on the other hand, increase activity time, food consumption rates, and reproductive rates, reducing abundance at lower trophic levels and favoring top‐heavy chains (i.e., communities with higher biomass or species richness on higher trophic levels) and top‐down control (Danet et al., 2021; Romero et al., 2018; Welti, Kuczynski, et al., 2020; see Figure 1 (III) and Table 1c). Additionally, predators are more sensitive to climate changes than primary consumers because they have a higher habitat requirements, in fact, previous evidence demonstrated that predator with larger bodies were extinct in greater proportion in regions with higher climatic instability since the Last Maximum Glacial (Sandel et al., 2011; Voigt et al., 2003). Consequently, regions with lower climatic instability are a refuge to predators, resulting in an imbalance in the predator–prey relationships, increasing the dominance of some predator species with larger body size and favoring top‐heavy chains compared to regions with higher climatic instability. However, the bottom‐up control generally has higher importance to determine species richness patterns at the macroscale than top‐down control (Sandom et al., 2013). Thus, communities with bottom‐heavy chains tend to be more diverse than top‐heavy chains at global scales (Danet et al., 2021; Sandom et al., 2013; Figure 1 (V)).

In this study, we seek to integrate macroecological theory with metabolic ecology and food web theories to explain the direct (climate → species richness) and indirect (climate → trait → species richness) effects of the contemporary climate and climate instability on the global richness of tetrapods (Figure S1). Specifically, we test the following predictions (detailed in Table 1):

1. Warmer and rainier regions with lower climatic instability have greater species richness for all tetrapod groups than colder and drier regions with higher climatic instability; this relationship will be stronger in amphibians and squamates than in birds and mammals (Figure 1 (I)).
2. Warmer and rainier regions with lower climatic instability have smaller birds and mammals, and larger amphibian and squamate species than colder and drier regions with higher climatic instability (Figure 1 (II)).
3. Greater size variation and top‐heavy pyramids of tetrapods are correlated with warmer and rainier regions with lower climatic instability (Figure 1 (III)).
4. Warmer and rainier regions with lower climatic instability have an indirect, positive effect on species richness by favoring body size reduction and increasing size variation. In contrast, these climates favor top‐heavy pyramids and indirectly reduce species richness (Figure 1 (VII) Unifying theories).

## 2 METHODS

### 2.1 Species distribution matrices

The distribution polygons for amphibians, birds, mammals, and squamates from the International Union for Conservation of Nature (IUCN, 2023) were utilized to calculate species richness per grid cell. We compiled the IUCN occurrence polygons for 15.788 tetrapod species containing 220 amphibians, 2.512 squamates, 7.949 birds, and 5.107 mammals. We standardized taxon‐specific distribution polygons to the same spatial resolution and projection (i.e., Mollweide projection) to create 2° grids (i.e., ~220 km) covering all terrestrial ecosystems in the globe. Then, we extracted the number of species on each grid and used these grids as the sampling unit in statistical analysis. Hereafter, we will refer to the species found on each grid as a “community” because this scale encompasses all species that potentially live and interact (Fauth et al., 1996).

### 2.2 Defining trophic structure and body size to calculate grid‐scale trait information

To gain a more realistic understanding of how climate influences species richness, we focus on two key community‐wide species traits: size structure and trophic structure. Community‐wide species traits represent the average trait shared by multiple species within a community (Ibarra‐Isassi et al., 2023). These traits reflect physiological characteristics, ecological interactions, and resource utilization. The median body size provides insights into the physiological limits and energy requirements of species in relation to climate (Brown et al., 2004). Additionally, body size variance captures the diverse range of body sizes within a community, facilitating niche differentiation and reducing competition for resources (Hopwood et al., 2016). On the other hand, trophic structure considers the feeding relationships and positions of organisms within the food chain, influencing energy flow and species interactions (Brown et al., 2004; Voigt et al., 2003). By studying these traits, we can uncover the mechanisms through which climate impacts species richness, while considering the physiological and ecological dynamics associated with resource use and requirements.

We obtained the body size and diet of species using specialized databases for amphibians (Oliveira et al., 2017), squamates (Bauman et al., 2018; Feldman et al., 2016; Meiri, 2018), birds (Wilman et al., 2014), and mammals (Faurby et al., 2018). We used the body mass in grams as a size measure that is comparable across all taxa because to each group the body size is measured in a different way. Thus, these databases considered: For amphibians, body size is the maximum adult body mass; for squamates is the conversion of snout–vent length (SVL) or total length (TL) to body mass by clade‐specific allometric equations; for mammals, the body size is the mass in species level and, while in the absence of data, body mass was estimated based on morphological correlates or phylogenetic imputation; and for birds, body size is geometric mean of average values of body mass provided for both sexes or mass–length relationships parameterized at family level.

We used the “Taxize” package and expertise of each group to standardize the taxonomic names between the IUCN and traits databases (Foster et al., 2018). After this, we removed species with missing information about body size and trophic level in traits databases. Our final occurrence database had 12.034 tetrapod species with trait information, corresponding to 76.2% of the IUCN database. Specifically, we used 178 amphibian species, 1.501 squamates, 6.607 birds, and 3.748 mammals in the analyses.

Body size was defined as the median value of body mass (in grams) of all species in a grid cell, whereas body size variation was calculated on each cell. The disparity in body sizes in a grid increases as body size variation increases. Additionally, we use the logarithm of median body size in the statistical models because body size has a skewed right distribution with higher predominance of lower body size species (Kozłowski & Gawelczyk, 2002).

We categorized species diets into three trophic levels to define the trophic structure at the community level: (i) primary consumers, which are represented by organisms that have more than 90% of their diet predominantly composed of leaves, flowers, seeds, and fruits; (ii) secondary consumers, representing organisms with a diet composed predominantly (>90%) of invertebrates and vertebrates; (iii) omnivores, organisms with a mixed diet without predominance of plant or animal components (<90%). After defining the trophic level of each species, we calculated trophic structure of communities across tetrapods group by estimating the predominance of higher trophic levels by grid and assigning a weight to each category: primary consumers received a weight of 2, omnivores 2.5, and secondary consumers 3 (Welti, Kuczynski, et al., 2020; Welti, Prather, et al., 2020). We defined this weight for each trophic level based on previous studies with N isotopes, which determine an intermediate position to omnivores relative to primary and secondary consumers (see Welti, Kuczynski, et al., 2020; Welti, Prather, et al., 2020). Furthermore, the trophic structure was estimated by the average trophic level weights of species of each tetrapod group that occur in each grid. Values close to 2 indicate a given grid has a bottom‐heavy pyramid with greater dominance of herbivores, whereas values close to 3 reflect a top‐heavy pyramid with greater dominance of predators and omnivores (McCauley et al., 2018; Welti, Kuczynski, et al., 2020).

### 2.3 Species richness

**FIGURE 2:**
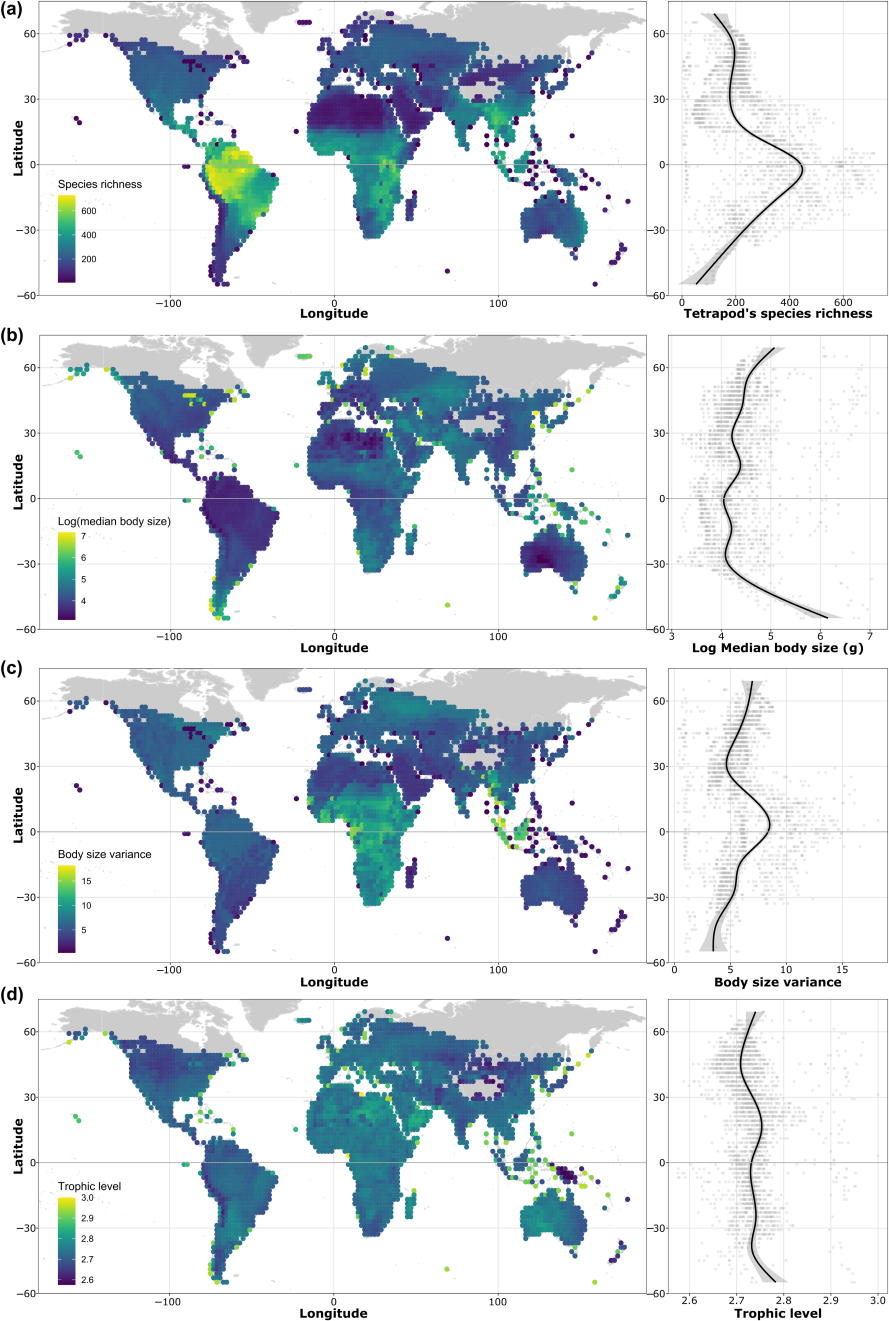
Latitudinal pattern of tetrapod (a) species richness, (b) body size, (c) body size variance, and (d) predominance of higher trophic levels.

We used the final occurrence data containing only species with traits information to calculate species richness per grid. The species richness was calculated by summing all species whose polygons intersect the center of each grid. We excluded grids with less than three species from statistical analyses to reduce the sampling bias. The final species matrix included 2722 cells covering each tetrapod group distributed across all continents (from 54° S to 69° N and 161° W to 178° E, covering an area of ~13,353 km of latitude and ~37,629 km of longitude) (Figure 2).

### 2.4 Environmental data

Climatic variables were extracted from WorldClim v.2.0 at a spatial resolution of 10 Arcmin (Fick & Hijmans, 2017). Initially, we created 2° grids to extract the contemporary annual mean temperature (Bio 1) and mean precipitation (Bio 12), using coordinates of grid centroids. Furthermore, we obtained each grid temperature and precipitation in the Last Glacial Maximum (~21,000 years BP) by using the MIROC‐ESM model. Temperature and precipitation anomalies are characterized by the difference between actual mean temperature (Bio 1) and mean precipitation (Bio 12) and from historical annual mean temperature or precipitation of last glacial maximum (e.g., García‐Andrade et al., 2021). When the difference between the current and historical values is close to zero, imply a lower temperature and precipitation anomaly, which indicates that the projected climatic change might be less pronounced or negligible in that region (Hortal et al., 2011). We emphasize that despite the uncertainties linked to models of climate reconstruction, it has been demonstrated that the inclusion of paleoclimatic information is fundamental to understanding current macroecological patterns (García‐Andrade et al., 2021; Hortal et al., 2011).

### 2.5 Composite variables

Composite variables are used to represent multivariate and complex theoretical concepts and their effect on response variables (Grace & Keeley, 2006; Santos et al., 2020). As a result, combining multiple operational variables into a single conceptual variable in the model reduces the number of interactions which, in turn, prevents an inflated model and allows a better interpretation of the relationships between the composite variables and the response variable (Grace & Keeley, 2006; Santos et al., 2020). We estimated the composite variables using a multiple regression between operational variables and response variables (see Appendix S1). Then, we multiplied the values of operational variables by their coefficient regression and summed them to estimate the values of composite variables to use in the structural models (Grace & Keeley, 2006).

**FIGURE 3:**
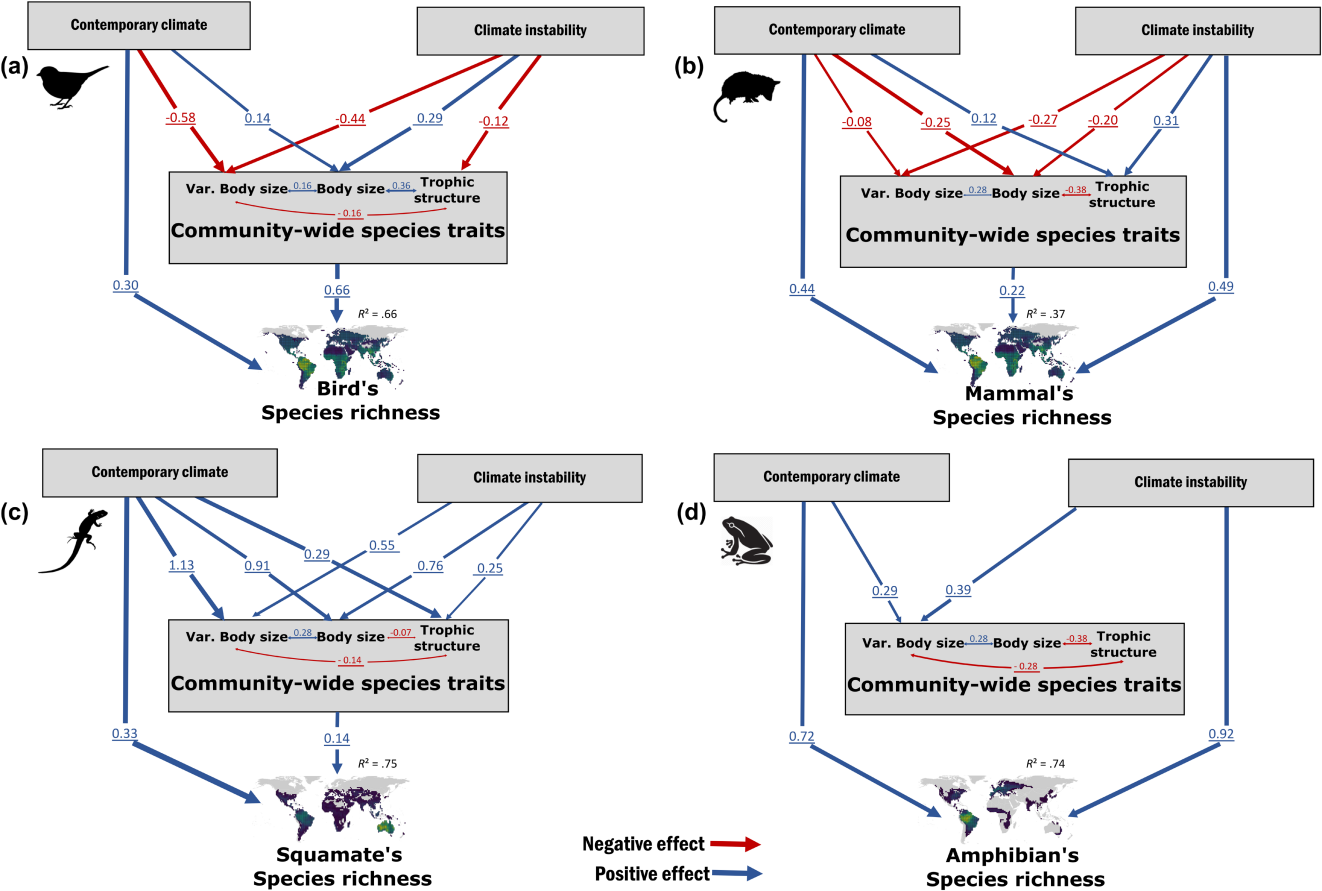
Structural model of piecewise structural equation model (pSEM) showing the relationship between the predictor and response variables emphasizing the direction and effect size on species richness to each tetrapod group, respectively, to (a) birds, (b) mammals, (c) squamates, and (d) amphibians. We represented only those significant relationships (*p* < .05). The blue and red colors represent, respectively, positive and negative relationships between the variables.

We used three composite variables: (1) contemporary climate, which is represented by combining the contemporary temperature and precipitation, with higher values indicating warmer and wetter regions (Figure S2); (2) climatic instability, which is composed of temperature and precipitation anomalies, with lower values representing regions with lower climatic variation since last maximum glacial; and (3) community‐wide species traits, composed of body size, size variation, and trophic structure that have a unique relationship to each group (Figure 3; Figure S3). As a result, the combined effect of the component operational variables on the response variable is represented by this new path through the composite variable: operational variables → composite variable → response variable. For example, the effect of contemporary temperature and precipitation on species richness is represented by the effect of a unique composite variable (i.e., contemporary climate) on the number of species.

### 2.6 Statistical analyses

We used the piecewise structural equation model (pSEM) to test how community‐wide species traits mediate the effects of contemporary climate averages and climate instability on species richness of each tetrapod group. Thus, we performed a pSEM containing the composite variables based on the theoretical model (Figure S1). We used separate pSEM in the following subgroups: birds, mammals, amphibians, and squamates. Before running the final pSEM model, we excluded the variables that were highly correlated (*r* > .7) based on the models with the lower Akaike information criterion (AIC) (see Figure S2, Appendix S1).

The pSEM models enable for the simultaneous testing of multiple hypotheses in which the variables act as response and explanatory variables, allowing for the partitioning of the total effects of the variables into direct (i.e., the relationship is not explained by another variable) and indirect effects (i.e., the relationship is explained by another variable) (Lefcheck, 2016). We defined contemporary climate (temperature and precipitation averages) and climate instability (temperature and precipitation anomaly) as exogenous variables, while community‐wide species traits act as endogenous variables to explain tetrapod richness; species richness can be directly affected by exogenous variables or indirectly by factoring out the link between exogenous and endogenous variables (Figure S1). This model also allows the inclusion of the correlation between endogenous variables.

Furthermore, each pSEM was composed of four ordinary least squares (OLS) that assess: (1) effects of contemporary climate, climate instability, and community‐wide species traits per grid on tetrapod species richness and (2) effects of contemporary climate and climate instability on the (a) trophic structure, (b) on species body size, and (c) on species size variation. In addition, we also considered the correlation between body size, size variation, and trophic structure on composite variable community‐wide species traits (see Appendix S1). We also performed a “Distance‐based Moran's eigenvector analysis” to obtain the eigenvectors (MEMs) representing the shortest distance connecting the locations with the highest autocorrelation (Dray et al., 2012). By including the MEMs in the models, we were able to explicitly estimate the effects of the spatial autocorrelation on the results. We first performed the OLS analysis for each response variable. Then, we extracted the residuals to select the MEMs to be added in each model, thus we minimized the autocorrelation in the residuals (MIR method‐Bauman et al., 2018). Later, we used OLS adding the spatial vectors (MEMs) to evaluate the spatial autocorrelation of each model with Moran's *I* that presented values lower than 0.8 (Table S1). Thus, we deal with the trade‐off between minimizing spatial autocorrelation and inflating the model by including a greater number of eigenvectors (Lefcheck, 2016; Santos et al., 2020). Although there is still autocorrelation in the models, we have minimized the effect of spatial autocorrelation on variables that are highly spatially structured. Finally, we used the four OLS models adding selected MEMs as structural models of our pSEM to each tetrapod group. The pSEM was performed using the R “piecewiseSEM” package (Lefcheck, 2016). All analyzes were performed using the R 3.5 software (R Core Team, 2019).

The strength and direction of direct and indirect effects were interpreted based on the standardized effect size of each link between two variables. The indirect effects of the exogenous variables were obtained from the multiplication of the direct effects (García‐Andrade et al., 2021; Lefcheck, 2016). To understand the role of contemporary climate, climate instability, and community‐wide species traits on species richness, we summed the direct and indirect effects of each variable to individually obtain each total effect (see Appendix S1). However, we point that the random variation attributed to species traits variables may be a result of phylogenetic relatedness, but because our analyses are conducted at the assemblage‐level, this effect is reduced, although species traits do exhibit some phylogenetic signal.

## 3 RESULTS

We found a latitudinal pattern in the distribution of species and traits of tetrapods, with regions closer to the equator (e.i., regions with higher contemporary climate and lower climate instability, see Figure S4) having more species, more variation in body size, and species with smaller body sizes than those farther away from the equator (Figure 2a–c). For example, moving from 10° of latitude to the equator increases the average species richness and body size variation, respectively, by 9.7% and 0.3%, and decreases body size by 2.2% for all tetrapods. However, there is no clear latitudinal pattern in trophic structure, suggesting a similar proportion of primary consumers, secondary consumers, and omnivores in tropical and temperate regions (Figure 2d).

Although the latitudinal pattern of species richness is similar for all groups, there are distinct patterns to traits across tetrapod groups (Figure S5). Specifically, birds and mammals tend to be smaller, while squamates are larger. In these groups, there is also higher size variation next to the equator and has no clear latitudinal pattern to trophic structure. On the other hand, the amphibians have no clear pattern to body size and size variation but tend to have more top‐heavy structures on temperate regions. We also could observe that there is predominance of omnivores to birds and mammals, but the secondary consumers are predominant to amphibians and squamates (Figure S6). Thus, bird and mammal communities tend to be more bottom‐heavy than amphibians and squamates communities.

Contemporary climate, climate instability, and community‐wide species traits explained, on average, 69% of the variation in species richness to each tetrapod group (details of each model on Table S1). The contemporary climate (temperature and precipitation average) and climate instability (temperature and precipitation anomaly) showed a positive relationship with species richness to mammals and amphibians, but only to contemporary climate is related to bird and squamate species richness. Still, this effect was stronger in amphibians and squamates (Figure 3a–d). We also found a positive correlation between community‐wide species traits with species richness for all groups, except for amphibians. In addition, communities with higher species richness are mostly associated with smaller bodies and more bottom‐heavy structures in birds and smaller bodies in mammals and squamates. Thus, we showed distinct routes in which contemporary climate and climate instability affect species richness through the community‐wide species traits.

### 3.1 Direct effects of climate on species richness

**FIGURE 4:**
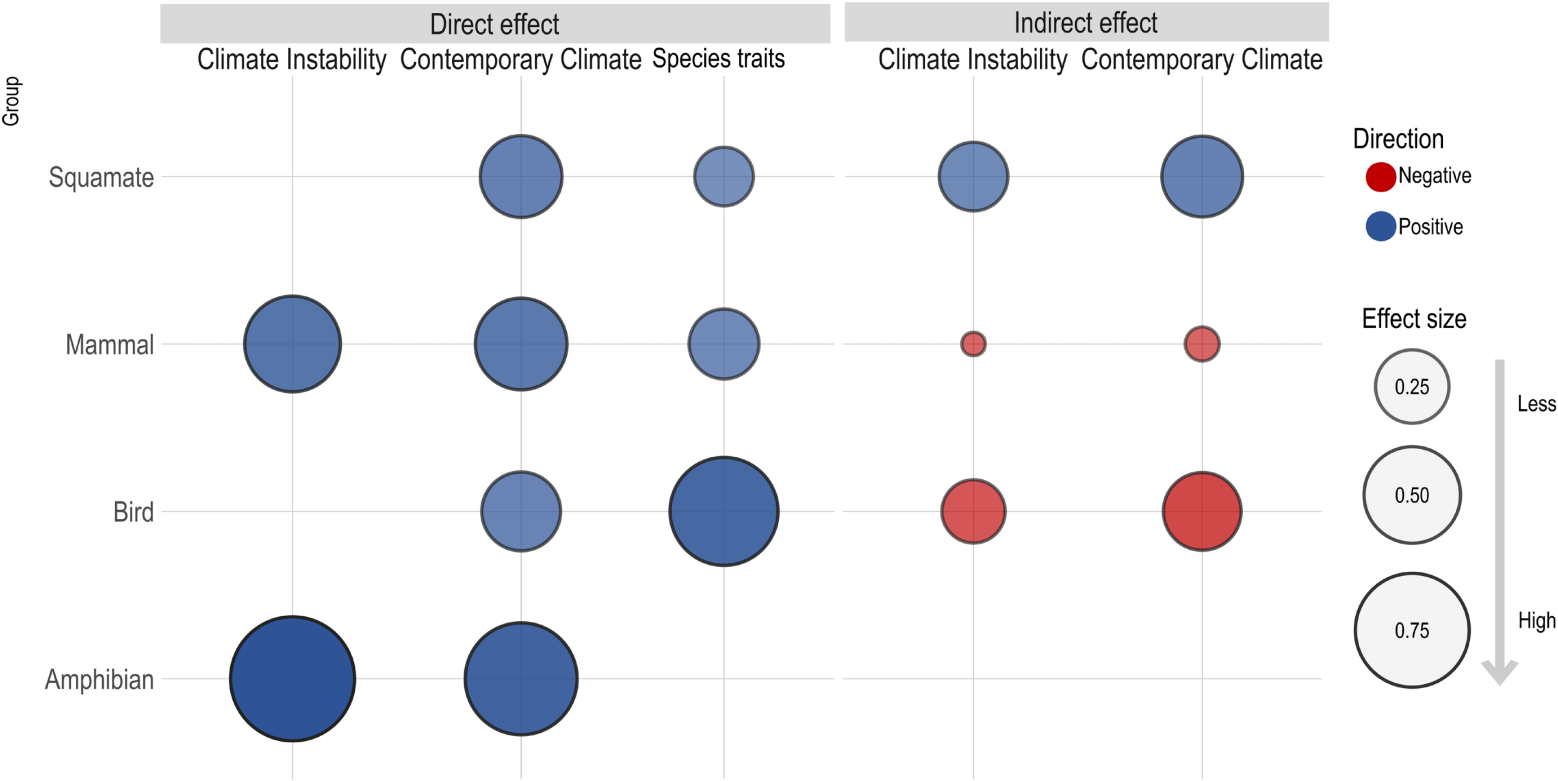
Effect size of composite variables (i.e., contemporary climate, climate instability, and species traits) on species richness of each tetrapod group. Fill color represents positive (blue) and negative (red) relationships between variables and circle size represents the strength of these relationships (standardize estimates).

We observed that regions with higher temperature, precipitation, and higher climatic instability have more tetrapod species (Table S2). However, the contemporary climate and climate instability have different relative importance to each group (Figures 3 and 4). Bird and mammal richness response to contemporary climate and climate instability is comparatively lower than that of amphibians and squamates. Nonetheless, bird and squamate species richness is only affected by contemporary climate, while amphibians and mammals are determined by both contemporary climate and climate instability. More specifically, contemporary climate and climate instability have similar importance to mammals, but amphibians species richness is mostly influenced by climate instability (Table S2).

### 3.2 Correlation between climate and community‐wide species traits

**FIGURE 5:**
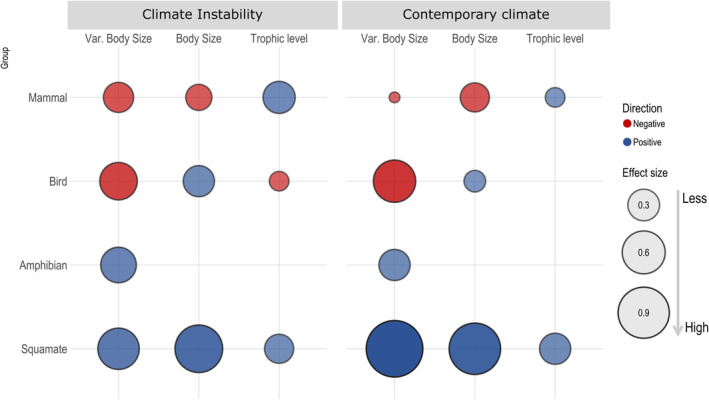
Effect size of composite variables (i.e., contemporary climate and climate instability) on species traits that compose the composite variable species traits of each taxon (i.e., body size, body size variance, and trophic structure). Fill color represents positive (blue) and negative (red) relationships between variables and circle size represents the strength of these relationships.

Likewise, contemporary climate and climate instability affected species richness and community‐wide species traits, but their relative importance varied among tetrapod groups (Figures 3 and 4). Warmer and rainy regions have bird species with larger body sizes (standardized *β* = .14) and less size variation (standardized *β* = −.58) than in colder and drier regions. Additionally, higher climatic instability has species with higher body sizes (standardized *β* = .29) and less body variation (standardized *β* = −.44) (Figures 3a and 5). Thus, the contemporary climate and climate instability acting in a synergistic way to favor larger‐bodied birds and lower size variation in warmer, wetter, and higher climatic instability. Nevertheless, contemporary climate is the main drive to body size, while climate instability determines the body size variation (Table S2). Moreover, climate instability is the only determinant of the trophic structure, favoring bottom‐heavy pyramids in birds in higher climatic instability (*β* climate instability = −.12, *p* < .001, Figures 3a and 5; Table S2).

For mammals, we demonstrated that the contemporary climate and climate instability have a combined effect on body size and trophic structure (Figures 3b and 5). Warmer and rainier regions with higher climatic instability have mammal species with smaller bodies, lower body size variance, and top‐heavy pyramids (Table S2). The contemporary climate (i.e., temperature and precipitation average) and climate instability has a similar negative effect on body size. Conversely, the influence of climate instability on body size variation and the trophic structure is around 2.8 times stronger than the contemporary climate (Table S2). Thus, climate instability outperforms the contemporary climate as a global driver of trophic structure and body size variation of mammals, while contemporary climate and climate instability has similar importance to determine the body size of mammals.

In warm and wetter regions with higher climatic instability, the squamate communities have top‐heavy pyramids, larger species, and higher size variation. Furthermore, contemporary climate and climate instability act synergistically favoring this size and trophic structure on squamates (Figure 3c). However, the contemporary climate is the primary driver of body size and size variation, although it has similar importance to climate instability in determining trophic structure (Table S2). Finally, we find that community‐wide species traits of amphibians are less influenced by climate than other tetrapods, with their body size variance being the only dependent variable influenced by climate (Figures 3 and 5; Table S2). The increase in temperature, humidity, and climate instability favors more body size variance in amphibians (Figure 3d).

### 3.3 Climate plays a role in the correlation between species richness and community‐wide species trait

We found that community‐wide species traits were correlated with higher species richness, but the strength and way of this relationship varied among tetrapod groups. For example, the species richness of birds is favored by community‐wide species traits around four times more than that of other tetrapod groups. Therefore, smaller‐bodied species and bottom‐heavy structures are likely to be associated with higher species richness (Figure S3). In similar ways, squamate communities with smaller species tend to have greater richness, but mammal species richness is favored mainly by top‐heavy structures. However, the direct route between species traits (body size and trophic structure) at the community level did not influence the number of amphibian species. Therefore, despite the community‐wide species traits favoring species richness on birds, mammals, and squamates, these groups have distinct size and trophic structure.

We demonstrated that the contemporary climate and climate instability indirectly reduce the bird and mammal richness through community‐wide species traits. Notably, these indirect pathways differ between birds and mammals communities (Figure 4). We found that warmer, wetter climates and higher climatic instability indirectly reduce the bird species richness because they favor similar size between the species and bottom‐heavy communities. However, these climates have a weak positive effect on bird species richness through increasing the body size. About mammals, we showed that indirect reduction in species richness occurs due to the stronger effect of reduction on body size and size variation by contemporary climate and climate instability, despite a top‐heavy structure indirectly favoring species richness.

On the one hand, we found that the contemporary climate and climate instability indirectly increases the species richness through community‐wide species traits in squamates, and it has no effect on amphibians (Figure 4). On squamates communities, higher contemporary climate and climate instability favor larger‐bodied species, higher size variation, and top‐heavy structures, consequently, indirectly increasing the species richness. Furthermore, body size, size variation, and trophic structure are mediators of the contemporary climate and climate instability effects on birds, mammals, and squamates, but they have no indirect effect on amphibian species richness.

## 4 DISCUSSION

Our study is the first one demonstrating that community‐wide species traits mediate the effects of contemporary climate and climate instability on global species richness, which emphasizes the importance of integrating climate‐based hypotheses into a more mechanistic framework in macroecology (Baiser et al., 2019; McGill, 2019). We first predicted that warmer and rainier regions with lower climatic instability have greater species richness for all tetrapod groups, but the climate effect could be stronger in amphibians and squamates than in birds and mammals. As expected, warmer and wetter regions have greater species richness for all tetrapod groups. However, we revealed that regions with higher climatic instability generally have higher species richness of mammals and amphibians. We also found that amphibians and squamates species richness are more affected by contemporary climate and climate instability than birds and mammals, supporting our prediction 1. Moreover, we observed that different traits at the community scale determine the response of tetrapod groups to contemporary climate and climate instability. More specifically, smaller mammals, larger squamates, and birds were associated with warmer and wetter climates with lower climatic instability. However, the body size of amphibians did not correlate with climate. This outcome contradicts our prediction 2 for birds but supports it for mammals and squamates. Additionally, in these regions, birds and mammals exhibit similarity in body size. However, there is lower similarity in body size for squamates and amphibians. This result is partially in line with our third prediction. Top‐heavy pyramids for mammals and squamates are more favored in warmer and wetter climates with lower climatic instability, whereas bottom‐heavy pyramids for birds are more prevalent. Finally, we showed that contemporary climate and climate instability on species richness through community‐wide species traits have distinct direction, strength, and route to all tetrapod groups, except amphibians that there is no indirect effect. The observed pattern may be explained by limited data availability, which highlights the importance of further research. Therefore, it is urgent that a new investigation is performed to better understand the relationship between climate, traits, and amphibian biodiversity (see, e.g., Guirguis et al., 2023).

### 4.1 Direct effects of climate on tetrapod richness

Species richness of all tetrapod groups respond to contemporary climate, but only mammals and amphibian species richness directly respond to climate instability. Overall, regions with higher temperatures present greater thermal and kinetic energy resulting in higher speciation rates, whereas higher rainfall is associated with greater resource availability, allowing larger populations and lower extinction rates (Brown et al., 2004; Gillman & Wright, 2014). These results support the metabolic theory, which predicts warmer and wetter regions have greater species richness due to higher resource and energy availability, and consequently, greater survival under these conditions (Brown et al., 2004; Currie et al., 2004; O'Brien, 2006).

On the other hand, contrary to expected, we found that regions with higher climatic instability tend to be more diverse than regions with lower climatic instability to mammals and amphibians, while birds and squamates are unaffected by climate instability. These regions with higher climatic instability tend to promote new habitats or expand existing ones, becoming certain areas suitable for new species, and promote evolutionary adaptation and speciation to better adapt to the new environment increasing the species richness (Carnaval et al., 2009; Rangel et al., 2018). This result aligns with the hypothesis of ecological opportunity demonstrating how climatic instability can create opportunities for rapid speciation allowing a better understanding of the evolution of the latitudinal gradient (see Schluter, 2016). Thus, climate instability can favor higher species richness through the creation of new habitats, the promotion of evolutionary adaptation, and speciation that could cause significant changes on latitudinal patterns over time. These results contradict the prevailing hypothesis of climate stability, which suggests that regions with lower climate instability harbor more diversity due to reduced extinction rates and increased speciation (Fine, 2015). This difference between our findings and previous studies may be explained by two reasons: first, most studies did not directly assess climate instability or do not assess it simultaneously with the contemporary climate.

The bird and mammal species richness showed a response weaker to the contemporary climate and climate instability compared to squamates and amphibians, thereby lending support there are asymmetric relationship of climate and global species richness between ectotherms and endotherms (Barreto et al., 2021; Marin et al., 2018). The greatest response of squamates and amphibians richness to climate may be related to a narrow thermotolerance range and lower dispersal ability compared to mammals and birds, which results in a higher geographical turnover (Buckley et al., 2012; Ficetola et al., 2021). Moreover, we found that climate instability is a better predictor than contemporary climate to amphibians and both climates has similar strength to mammals, while the contemporary climate is the unique predictor to birds and squamates. Importantly, as current macroecological theory frequently uses patterns observed in endotherms to generalize to ectotherms (see Pincheira‐Donoso et al., 2021), our findings demonstrate that endotherms and ectotherms have distinct macroecological patterns and provide an enhanced mechanism for explain the macroecology of ectotherms. In addition, our results reinforce the importance of climate instability to predict the species richness mainly of bad‐disperser and narrow‐ranging groups (Araújo et al., 2008). For example, birds' higher dispersal capacity allows them to move between regions and track appropriate climates to avoid climate changes (Buckley et al., 2012; Ficetola et al., 2021; Jetz et al., 2007). In contrast, mammals and amphibians are more constrained by geographical barriers, making them the most sensitive tetrapod to climate instability (Ficetola et al., 2021). Additionally, squamates have a narrow term tolerance and low dispersal capacity than birds might also become constrained by geographic barriers due to the similarity of their response to that of amphibians (Araújo et al., 2013; Buckley et al., 2012). Taken together, these results emphasize the importance of ecological traits (e.g., body size and dispersal ability) and metabolic factors (e.g., heat tolerance) to understand the correlation between climate instability and species richness patterns at the global scale.

### 4.2 Correlations between climate and community‐wide species traits improve predictions of species richness at large scales

We found that the contemporary climate and climate instability have different correlation patterns with size structure (i.e., median and variance) of tetrapods. Thus, warmer and wetter climates with higher climatic instability have smaller mammals, but larger squamates and birds, than colder and drier regions. However, they do not affect amphibian's body size. Our findings support the Bergmann's rule for mammals (Meiri & Dayan, 2003), the reverse pattern for squamates (Ashton & Feldman, 2003), no support for amphibians (Adams & Church, 2008; Johnson et al., 2023), although it is opposite the expected pattern for birds (Meiri & Dayan, 2003; Salewski & Watt, 2017). These nuances of Bergmann's rule are highly debated and other explanations for Bergmann‐type clines, like precipitation, primary plant productivity, trophic level, and competition, have a key role acting simultaneously with temperature to determine the latitudinal body size pattern (Alhajeri & Steppan, 2016; Hantak et al., 2021). Furthermore, the contrasting result observed in birds may stem from the possibility that previous studies did not account multiple climatic variables and traits, including body size variation and trophic structure. This could lead to a bias in assessing the effect of climate on body size. Moreover, we showed that higher contemporary climate (i.e, higher temperature and precipitation) on lower latitudes are correlated with larger birds, but similar body size, and predominance of lower trophic levels. We also highlight that the use of only the mean or median of body size is limited, because the mean is biased due to the influence of extreme values that are more present at lower latitudes because of higher body size variation (Figure 2c). Moreover, the use of only the median of body size reduces the influence of extreme values but does not represent the variation in body size of the species. Furthermore, we reinforce the importance of simultaneous use of the median and variation of body size (i.e., size structure) to allow an unbiased and mechanistic understanding of the response of the community to climate (see Appendix S2).

The decrease of body size of mammals in regions with lower climatic instability could be due to smaller species being able to withstand climate change for longer periods than larger species, as expected by the metabolic theory (Gardner et al., 2011; Peralta‐Maraver & Rezende, 2021). Previous studies have shown that larger species are more affected by the warming climate than smaller species, mainly via changes in the length of their reproductive or feeding season (Gardner et al., 2011). However, we also found that birds and squamates tend to be larger bodied in regions with lower climatic instability that due these groups have different dispersal abilities and physiological tolerance. For instance, birds that are better at dispersing than other tetrapod groups could have their body size more determined by higher resource availability on regions with lower climatic instability than climatic tolerance (Ficetola et al., 2021). Nonetheless, the larger‐bodied squamates in regions with lower climatic instability could be due higher climatic tolerance of ectotherms than allow higher survival to climatic changes (Buckley et al., 2012; Ficetola et al., 2021). Moreover, we also found that these relationships between climate and body size indirectly favor higher species richness in communities with smaller mammals, and larger birds and squamates, supporting the “species packing” hypothesis (Ritchie & Olff, 1999). This hypothesis predicts that smaller species require lower resources and space and may have lower specialized ecological requirements, consequently, allowing higher coexistence in a given area (Ritchie & Olff, 1999). Furthermore, the negative relationship between body size and richness suggests that climatic‐mediated reductions in species body size favor resource partitioning, which drives macroecological diversity patterns.

Contrary to our expectations, birds and mammals have lower size variations in warmer and wetter climates with higher climatic instability, consequently decreasing the species richness. Higher temperatures and precipitation may reduce the interspecific competition of birds and mammals due to greater food availability allowing similar‐sized species to coexist (Buckley et al., 2012; Hopwood et al., 2016). Additionally, bodies tend to converge to a similar size in higher climatic instability climates, increasing the survival rate (Araújo et al., 2013; Phillips & Heath, 1995). However, the relevant climate variables driving size variation are distinct between birds and mammals. The contemporary climate is the main determinant of bird size variation, while climate instability is more important to mammals. This distinct importance of contemporary climate and climate instability can result from the lower dispersal ability of mammals than birds to avoid climate instability (Ficetola et al., 2021). Moreover, we found that this reduction of size variation by contemporary climate and climate instability has indirectly reduced the species richness of birds and mammals. This reduction of species richness occurs due higher similarity of body size increasing the niche overlap and competition, consequently, reducing the number of coexisting species (Hopwood et al., 2016).

We found that warmer and wetter regions have a remarkable squamate and amphibian size variation and these regions have higher coexistence of species. In warmer regions, squamate and amphibian species experience higher competition due to elevated temperatures favoring more time activity and larger species, but the simultaneous presence of higher precipitation increases availability of resources (Buckley et al., 2012; Hopwood et al., 2016). To squamates, the higher size variation could be linked to occupying distinct niches, accessing underutilized resources, and reducing competition intensity. Additionally, we also found that warmer and wetter climates with lower climatic instability are indirectly associated with increased squamate species richness through higher size variation. Thus, these results show that greater size variation to avoid competition is a primary drive to favor the species richness, despite that it is expected a higher interspecific competition reducing coexistence on squamate (Buckley et al., 2012; Peralta‐Maraver & Rezende, 2021). On the other hand, despite the climate favoring the size variation on amphibians, there is no effect on species richness. This absence of effect could be due to narrow variance of body size and predominance of predators on amphibian communities.

Lastly, the contemporary climate and climate instability are correlated with top‐heavy pyramids to mammals and squamates but bottom‐heavy to birds. Warmer and wetter regions feature pyramids of mammals and squamates that tend to be top‐heavy, as predicted by the food‐web theory (Welti, Kuczynski, et al., 2020). Despite corroborating this to mammals and squamates, we found no correlation between climate and trophic structure of amphibians that suggests this trend described in literature may be a bias of change on other variables, such as the variance in body size that favor top‐heavy pyramids when have lower size variation. Moreover, the association between higher climatic instability climates and top‐heavy pyramids of mammals and squamates may be due to decreasing plant abundance since the last glacial maximum, which reduces resource availability mainly to herbivores than omnivores and predators (McCauley et al., 2018). We found that top‐heavy pyramids tend to have lower bird species richness but, contrary to expectations, have greater mammal and squamates species richness. To birds, these results support the hypothesis that the top‐heavy trophic structure has lower species richness due to greater predation force that reduces the species abundance and consequently, species richness in lower trophic levels (Brown et al., 2004; Romero et al., 2018; Welti, Kuczynski, et al., 2020). However, the increase of mammal and squamate species richness in top‐heavy pyramids can be due to these groups having the main predators or higher abundance of predatory species, consequently, have higher top‐down control reducing the dominance of some herbivore species (Estes et al., 2011). Furthermore, we showed that warmer and wetter climates with higher climatic instability reduce bird species richness but favors mammal and squamate species richness through top‐heavy pyramids.

## 5 CONCLUSION

In this study, we provided a macroecological synthesis that integrates multiple hypotheses linking direct and indirect pathways by which contemporary climate and climate instability determine in complex and different ways the global distribution of birds, mammals, amphibians, and squamates. We provide a more detailed and mechanistic explanation supporting that endotherms and ectotherms have distinct macroecological patterns and response to climate, each influenced by different traits, allowing a better understanding of the current pattern of species distribution and the refinement of the risk of extinction due to climate change. Although the contemporary climate directly determines the species richness of all tetrapod groups, climate instability only influences bird and squamate species richness through community‐wide species traits. Additionally, we showed that contemporary climate and instability indirectly affect all tetrapod species richness, except amphibians, by altering body size and trophic structure. Thus, we demonstrated that species traits can explain the asymmetric responses of tetrapod species to climate. Likewise, macroecological studies using traits at the community scale may be benefitted by using different trait facets such as size structure and multiple traits averages. Our findings demonstrate that unifying multiple theories improves our knowledge of large‐scale diversity patterns across taxonomic groups by allowing us to make realistic and mechanistic predictions, which can improve macroecological theory.

## AUTHOR CONTRIBUTIONS

**Reginaldo A. F. Gusmão:** Conceptualization (lead); data curation (lead); formal analysis (lead); methodology (lead); writing – original draft (lead); writing – review and editing (equal). **Geiziane Tessarolo:** Writing – review and editing (equal). **Ricardo Dobrovolski:** Writing – review and editing (equal). **Thiago Gonçalves‐Souza:** Conceptualization (lead); formal analysis (equal); writing – review and editing (equal).

### OPEN RESEARCH BADGES

This article has earned Open Data, Open Materials and Preregistered Research Design badges. Data, materials and the preregistered design and analysis plan are available at https://doi.org/10.5061/dryad.bzkh189f0.

## Supporting information


Data S1.

Data S2.

## Data Availability

All data and script are provided in submission as a Supporting Information and deposited on https://doi.org/10.5061/dryad.bzkh189f0. All data also can acess by this link: https://github.com/ReginaldoGusmao/Gusmao_et_al_2023_EE.
