# Supplementary material for: Body size and trophic structure explain global asymmetric response of tetrapod diversity to climate effects
Source: Ecol Evol. 2024 Feb 20;14(2):e11047. doi: 10.1002/ece3.11047 (PMC10877556; doi:10.1002/ece3.11047)
Supplement: Supplementary file 2 — Data S2. [file ECE3-14-e11047-s001.docx]

BODY SIZE AND TROPHIC STRUCTURE EXPLAIN GLOBAL ASYMMETRIC RESPONSE OF TETRAPOD DIVERSITY TO CLIMATE EFFECTS

**Short running title: Direct and indirect ecological drivers of global tetrapod diversity**

**SUPPORTING INFORMATION**

**Appendix**

Appendix S1. Detail of piecewise Structural Equation Model (pSEM).

**1. Composite variables**

The composite variables could be determined as the sum of the effect's values of individual variables, with each operational variable having a distinct weight (statistical composite) on the composite variable. The weight of each variable is based on the coefficient of an ordinary least square (OLS). Thus, we used an ordinary least square (OLS) to evaluate operational variables' effect on species richness. After this, the values of each operational variable were multiplied by their coefficients (weight) and summed to generate the factor scores (composite variables).

We conducted an OLS on each tetrapod group assessing the effect of temperature (Bio 1), precipitation (Bio 12), anomaly temperature, anomaly precipitation, mean trophic level, median body size, and variance of body size on species richness. We then developed each composite variable as follows:

1. Contemporary climate: We multiplied the coefficient of contemporary temperature (Bio1) and precipitation (Bio12) by their coefficients and summed them to obtain the factor score to use on the structural model.
2. Climate instability: We multiplied the coefficient of temperature and precipitation anomalies by their coefficients and summed them to obtain the factor score.
3. Species traits: We multiplied the coefficient of mean trophic level, median and variance body size by their coefficients and summed them to obtain the factor score that it used on the structural model.

**2. Selecting variables**

We evaluated the correlation between our operational and composite variables in each tetrapod group (Fig. S2). Thus, we used Spearman’s correlation to avoid correlation problems between the variables and avoid inflating the models. We removed variables with strong correlations (r> 0.7) and evaluated the models with and without these variables. We observed that all tetrapod groups have a substantial correlation (r< 0.7) between temperature and temperature anomaly (Fig. S2). As a result, we removed the temperature anomaly variables that have lower p values, and ran the OLS models again selecting the models that had lower Akaike Information Criterion (AIC) values for each tetrapod group. The models with temperature and anomalous temperature show lower AIC than models with simply temperature for all tetrapod groups, except for birds (see Tab. S3)

**3. Structural equation to Piecewise Structural Equation Model (pSEM)**

After selecting the variables, we defined our theoretical model (Fig. S1) of the effects of composite variables on species richness. Based on the theoretical model, we created four structural equations for pSEM. We used the Ordinary Least Square (OLS) to perform structural equations that evaluate the (1) effect of composite variables (i.e., contemporary climate, climate instability and community-wide species traits) on species richness; (2) the effect of contemporary climate and climate instability on the (a) trophic structure, (b) on species body size, and (c) on species size variation. We follow this structure to each tetrapod group (i.e., bird, mammals, amphibian, and squamate). Furthermore, each group has four OLS models, totaling 16 structural models (Table S2).

**4. Distance-based Moran's eigenvector analysis**

We used the “Distance-based Moran's eigenvector analysis” to reduce spatial autocorrelation. We performed each model and used the residuals (Shown above - Topic 3) to obtain the eigenvectors (MEMs) that represent the highest autocorrelation with the response variable (p<0.05) (MIR method- Dray *et al.*, 2012). Then, we added the select MEMs to the OLS models with the function ‘listw.select’ of the package adespatial and ran the models once again (see selected MEMs – Table S2). Next, we assessed the spatial autocorrelation of each model using Moran's I, which was calculated with the residuals from our models. To perform this analysis, we employed the 'lm.morantest' function from the 'spdep' package. This calculation involved utilizing a spatial weights matrix (W) that defines the spatial relationships among our grid and the residuals of each model. Our observation revealed that models with R² values below 0.8 (as indicated in Table S1 - Moran's I) exhibited yet a spatial autocorrelation.Thus, we reduce the spatial autocorrelation on response variables and avoid inflating our models by including more eigenvectors although there is still autocorrelation in the models, although some variable responses remain spatially structured (Lefcheck 2016; see Appendix 3).

1. **Piecewise Structural Equation Model (pSEM)**

We used the piecewise Structural Equation model (pSEM) to evaluate composite variables' direct and indirect effects on the species richness of each tetrapod group (Fig. S2 e Table S1). We performed one pSEM to each tetrapod group using the five structural equations with the selected MEMs (Shown above – Topic 3 and 4, Table S1). We used the function ‘psem’ of the package “piecewiseSEM”. Moreover, we added to our pSEM the correlation relationships between the body size, size variance, and trophic level to avoid inflating the model using the ‘%~~%’. After performing the pSEM, we used the directed separation test to evaluate the independence claims in our pSEM (‘dSep’ function), as shown in our theoretical model (Fig. S1). We present Ficher’s C and R² values of each pSEM on Table S1.

Direct and indirect effects were interpreted based on the standardized effect sizes that link the variables (Table S2). The strength and direction of direct effect is the standardized effect size of these relationships, and we represented only significant effects (p > 0.05) in Figure 3. The indirect effects were obtained from the multiplication of the direct effects on a specific route of pSEM (Lefcheck, 2016; García-Andrade *et al.*, 2021). For example, the indirect effect of contemporary climate on species richness through species traits (pSEM route = contemporary climate -> species traits -> species richness) was calculated by multiplication of standardized estimate of contemporary climate effect on species richness, species traits on species richness, and contemporary climate effect on specie traits. Besides that, to understand the role of contemporary climate, climate instability, and species traits on species richness, we summed the direct and indirect effects of each variable to obtain each total effect (Figure 4 and 5).

**Reference**

Dray, S., Pélissier, R., Couteron, P., Fortin, M.J., Legendre, P., Peres-Neto, P.R., Bellier, E., Bivand, R., Blanchet, F.G., de Cáceres, M., Dufour, A.B., Heegaard, E., Jombart, T., Munoz, F., Oksanen, J., Thioulouse, J. & Wagner, H.H. (2012) Community ecology in the age of multivariate multiscale spatial analysis. *Ecological Monographs*, **82**, 257–275.

García-Andrade, A.B., Carvajal-Quintero, J.D., Tedesco, P.A. & Villalobos, F. (2021) Evolutionary and environmental drivers of species richness in poeciliid fishes across the Americas. *Global Ecology and Biogeography*, **30**, 1245–1257.

Lefcheck, J.S. (2016) piecewiseSEM: Piecewise structural equation modelling in r for ecology, evolution, and systematics. *Methods in Ecology and Evolution*, **7**, 573–579.

Appendix S2. Complementary analysis to understand the importance of community-wide species traits on body size and climate relationship.

We conducted a supplementary analysis using piecewise Structural Equation Models (pSEM), focusing solely on the median body size as a mediator of climate effects. By doing this, we repeated all analyses on the bird dataset replacing composite-wide species traits with the median body size (see Methods, main text). Previous studies indicate that communities with larger birds tended to exhibit lower species richness (Evans *et al.*, 2005). We corroborated these results when analysis using only the body size (Fig. 1). Thus, we showed that higher contemporary climate and climate stability favor higher species richness through reducing body size. However, upon examining community-wide species traits, we observed that the variance in body size plays a pivotal role in counteracting the increase in median body size and higher trophic level (see results Fig. 3a). We also reinforce that our model with all traits (R = 0.66) has higher power than the body size model (R= 0.43) to explain the species richness pattern to birds. Furthermore, the use of community-wide species traits provides us with a more comprehensive understanding of how species traits mediate the impact of climate on species richness. Therefore, our results highlight the importance of not relying solely on a single trait, as doing so could lead to biased interpretations regarding how climate influences species richness. For a more detailed discussion, please refer to the section titled "Correlations between climate and community-wide species traits improve predictions of species richness at large scales" in our discussion.

Figure 1. Structural model of piecewise Structural Equation Model (pSEM) We showed the relationship between the predictor and response variables emphasizing the direction and effect size on bird species richness using only body size with mediator. We represented only those significant relationships (p < 0.05). The blue and red colors represent, respectively, positive and negative relationships between the variables. We also showed the effect size of composite variables (i.e., contemporary climate, climate instability, and median body size) on bird species richness. Fill color represents positive (blue) and negative (red) relationships between variables and circle size represents the strength of these relationships (standardize estimates).


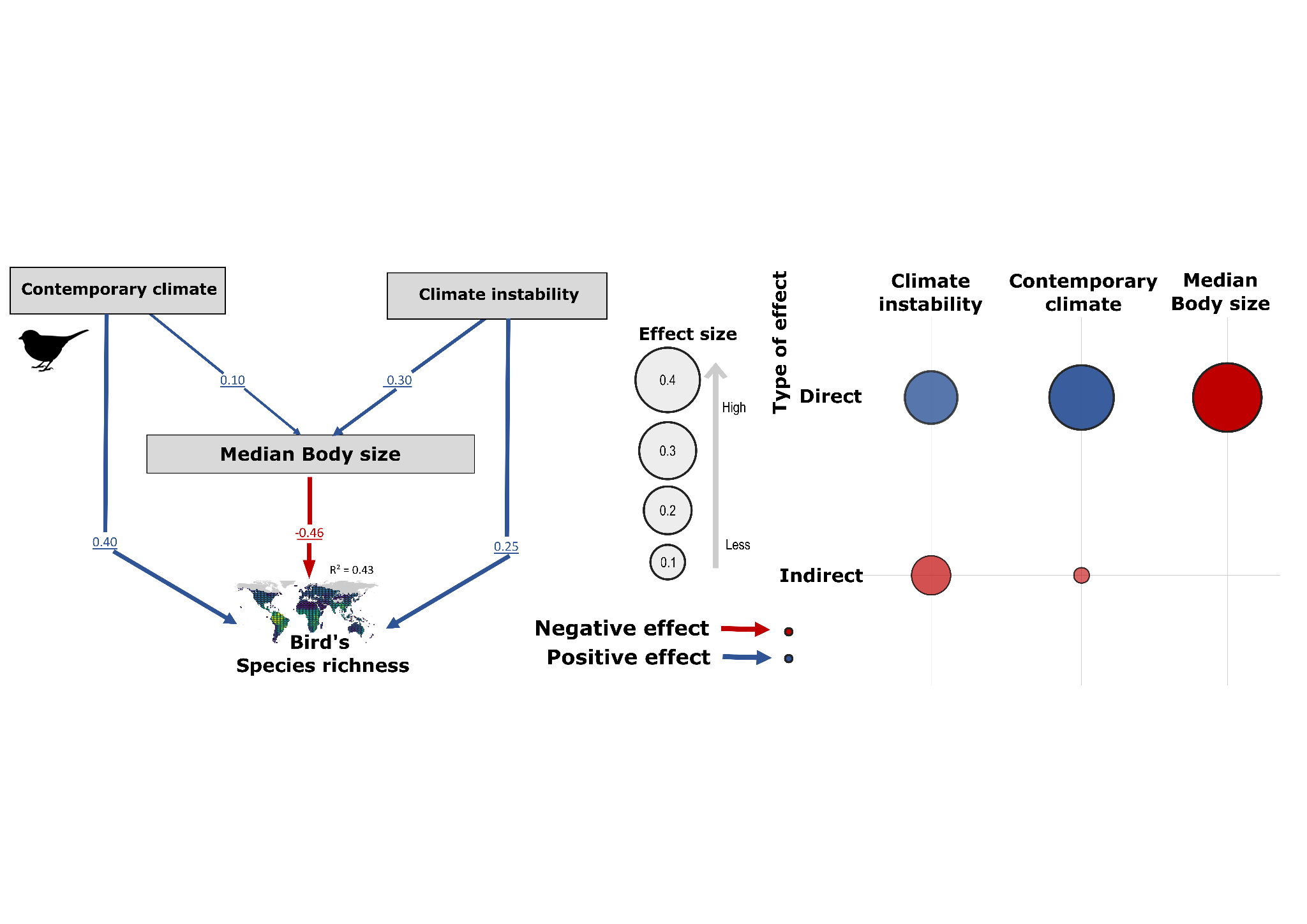


**Reference**

Evans, K.L., Warren, P.H. & Gaston, K.J. (2005) Species-energy relationships at the macroecological scale: A review of the mechanisms. Biological Reviews of the Cambridge Philosophical Society, 80, 1–25.

Appendix S3. Complementary analysis to understand the influence of spatial autocorrelation on results.

Although we use Ordinary least squares models (OLS) with “Distance-based Moran's eigenvector analysis” to minimize the influence of space on the results (see methods and Appendix S1), we observed that residuals were still spatially-structured (Tab. S1). Thus, we re-evaluated our models with the generalized least squares (GLS) analysis because it explicitly adjusts the models with correlated residuals using latitude and longitude (Pinheiro and Bates, 2000). Therefore, comparing our models with OLS + MEMs with GLS allows us to assess the robustness of the results despite the potential effects of the autocorrelated structure of the residuals. We found that similarly to OLS + MEMs, GLS was unable to fully control the spatial structure of the data across tetrapod groups (Fig. S3-1 to S3-4). This result indicates that there is a strong spatial structure that can slightly affect our models. It is important to emphasize, however, that OLS + MEMs models have higher R² and lower residuals than GLS models. We observed that OLS + MEMs and GLS models minimize in similar ways the autocorrelation to amphibians, squamates, and mammals data (Fig. S3-1 to S3-3), but OLS + MEMs models were better than GLS models to birds (Fig. 4). To make it consistent, we used OLS + MEMs analysis in the main manuscript because it is the most common method used in similar studies: Diniz-Filho *et. al*., 2009 and Santos *et. al.,* 2020).

Figure 1. Variogram to Ordinary least squares (OLS) with the Moran’s eigenvector maps (MEM) and to generalize least squares (GLS) models to amphibian data.

**
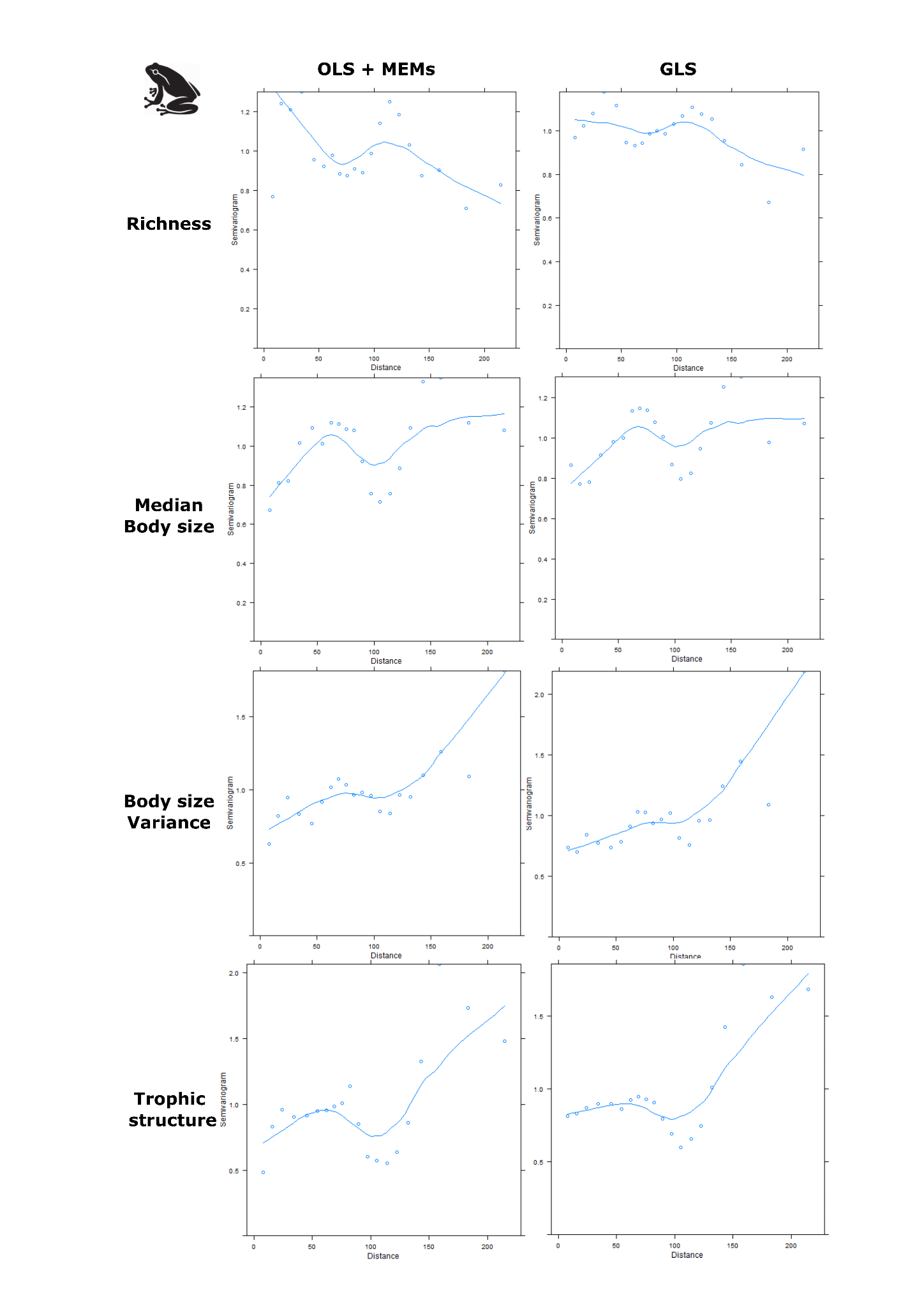
**

Figure 2. Variogram to Ordinary least squares (OLS) with the Moran’s eigenvector maps (MEM) and to generalize least squares (GLS) models to squamates data.

**
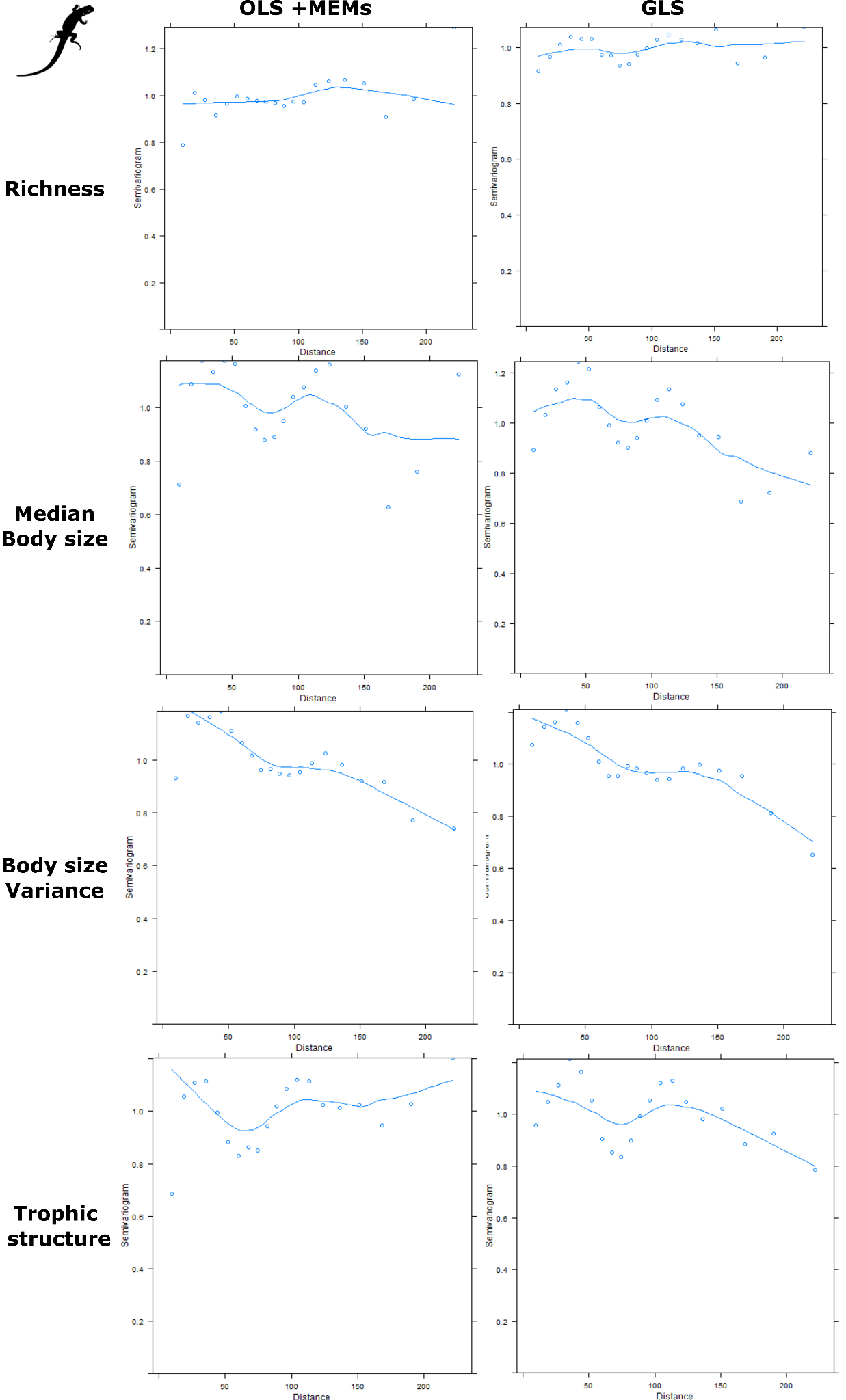
**

Figure 3. Variogram to Ordinary least squares (OLS) with the Moran’s eigenvector maps (MEM) and to Generalize least squares (GLS) models to mammals data.

**
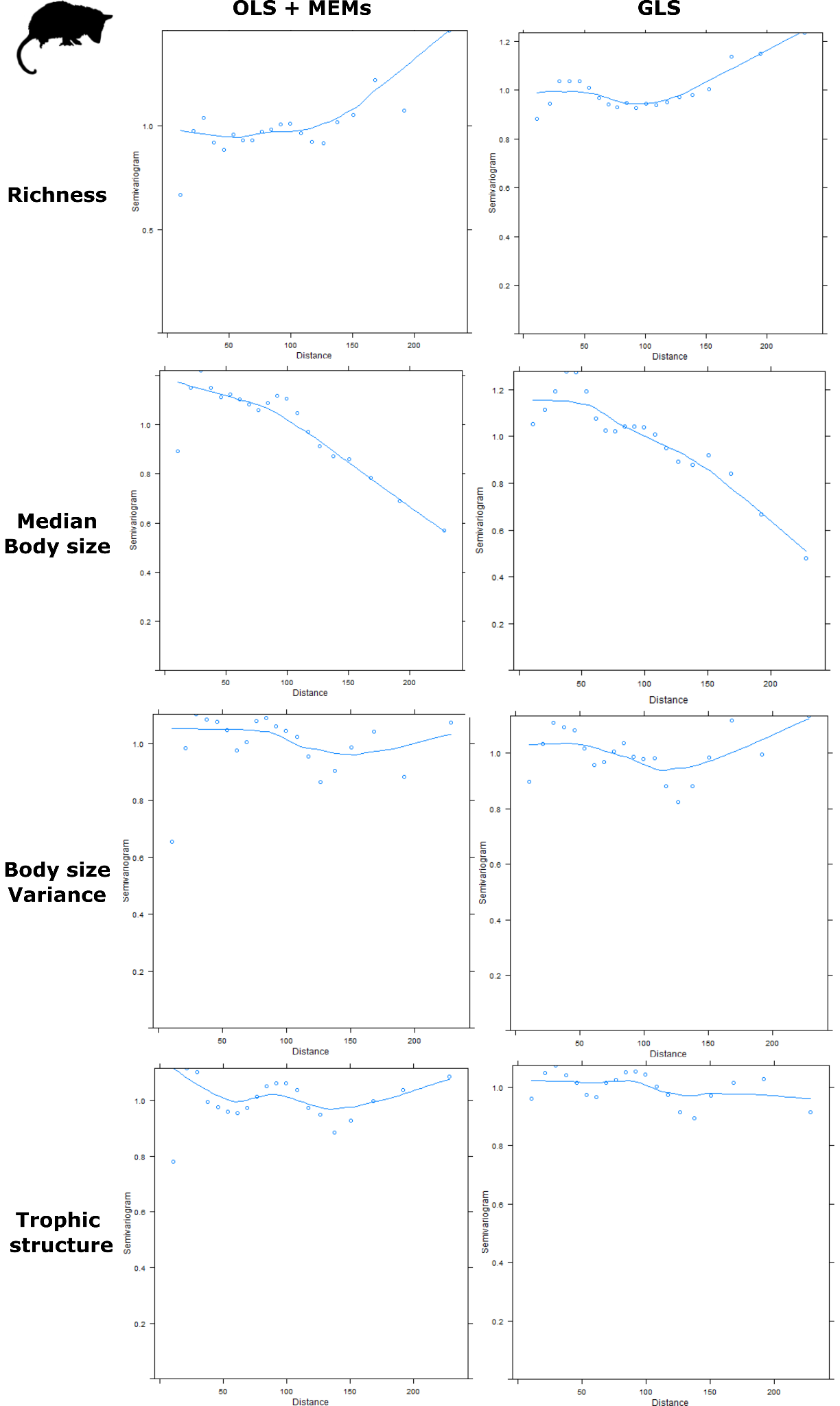
**

Figure 4. Variogram to Ordinary least squares (OLS) with the Moran’s eigenvector maps (MEM) and to Generalize least squares (GLS) models to birds data.

**
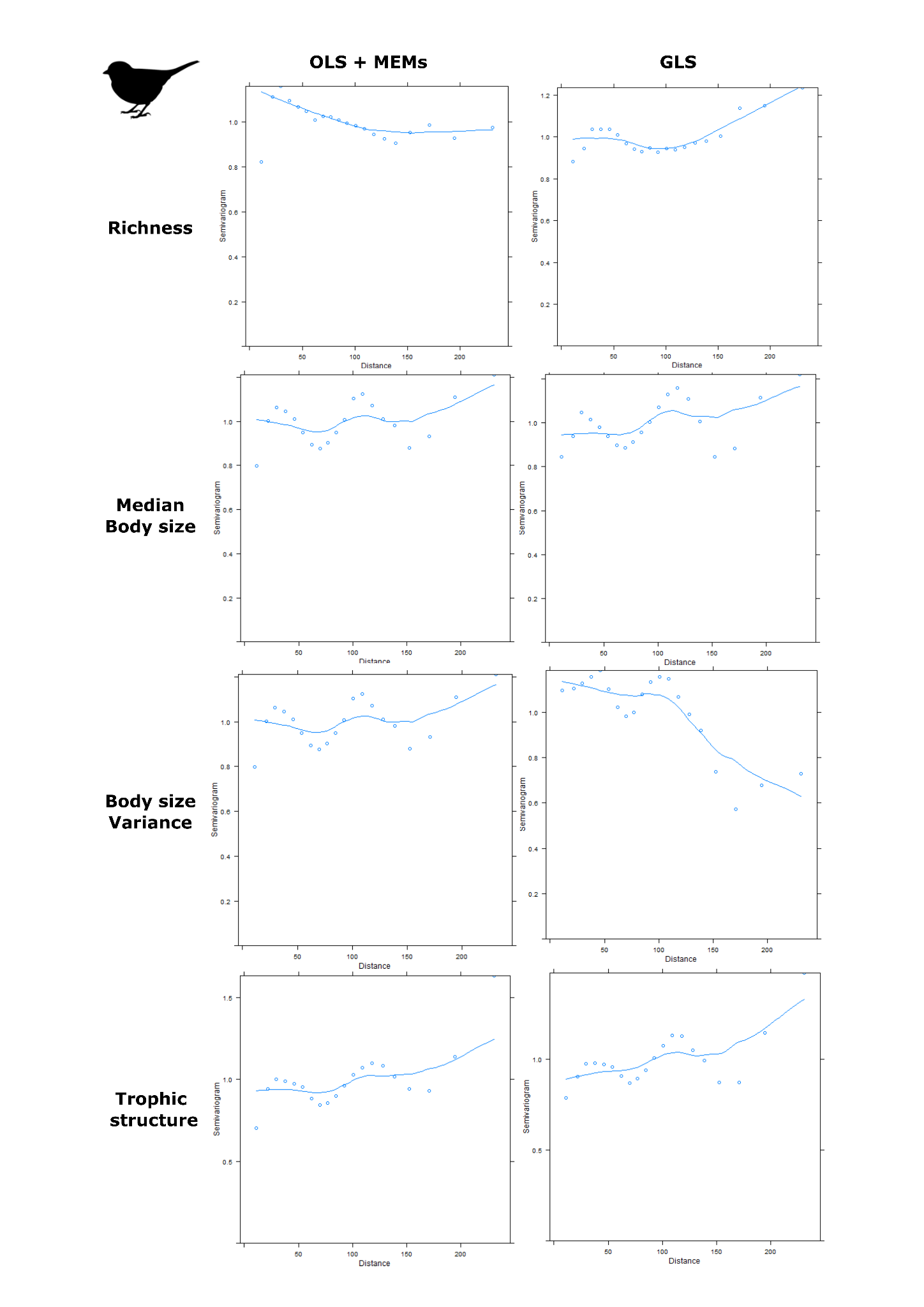
**

**Reference**

Diniz‐Filho, J. A. F., Rodríguez, M. Á., Bini, L. M., Olalla‐Tarraga, M. Á., Cardillo, M., Nabout, J. C., et al. (2009). Climate history, human impacts and global body size of Carnivora (Mammalia: Eutheria) at multiple evolutionary scales. Journal of Biogeography 36, 2222–2236. doi: 10.1111/j.1365-2699.2009.02163.x.

Santos, A. M. C., Cianciaruso, M. V., Barbosa, A. M., Bini, L. M., Diniz‐Filho, J. A. F., Faleiro, F. V., et al. (2020). Current climate, but also long‐term climate changes and human impacts, determine the geographic distribution of European mammal diversity. Global Ecol. Biogeogr. 29, 1758–1769. doi: 10.1111/geb.13148.

**Figures**

Figure S1. Theoretical structural model used in *piecewise Structural Equation Model* (pSEM). The expected relationships between exogenous (i.e., contemporary climate and climate instability) and endogenous variables (i.e., species traits) were used to understand their effect on species richness. The colors represent positive (blue) and negative (red) relationships between the variables.


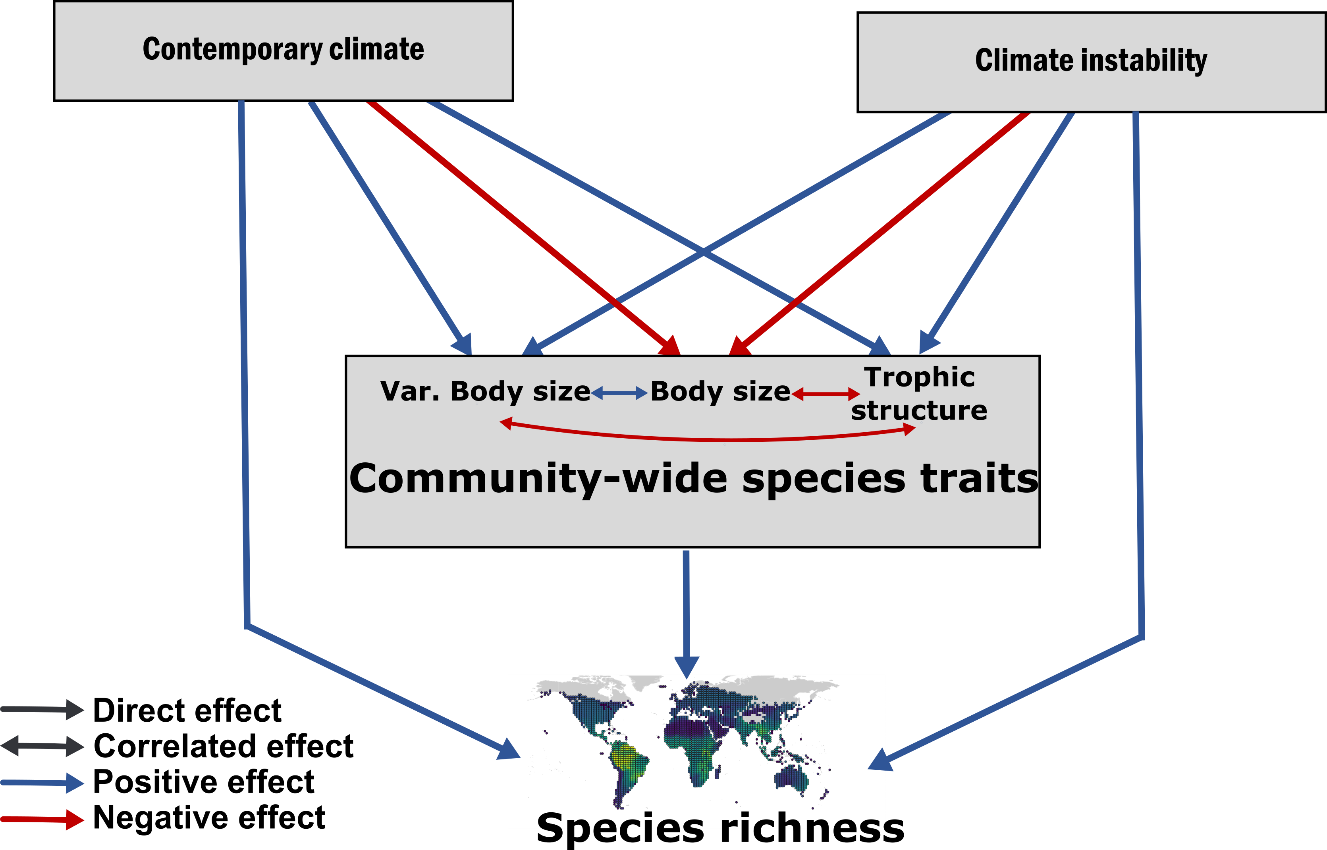


Figure S2. Correlation between the predictor variables used to structure the models. We avoided using a correlation higher than 0.7 between component variables (Green) and composite variables (Black) to each taxon: (A) bird, (B) mammal, (C) squamate, and (D) amphibian.


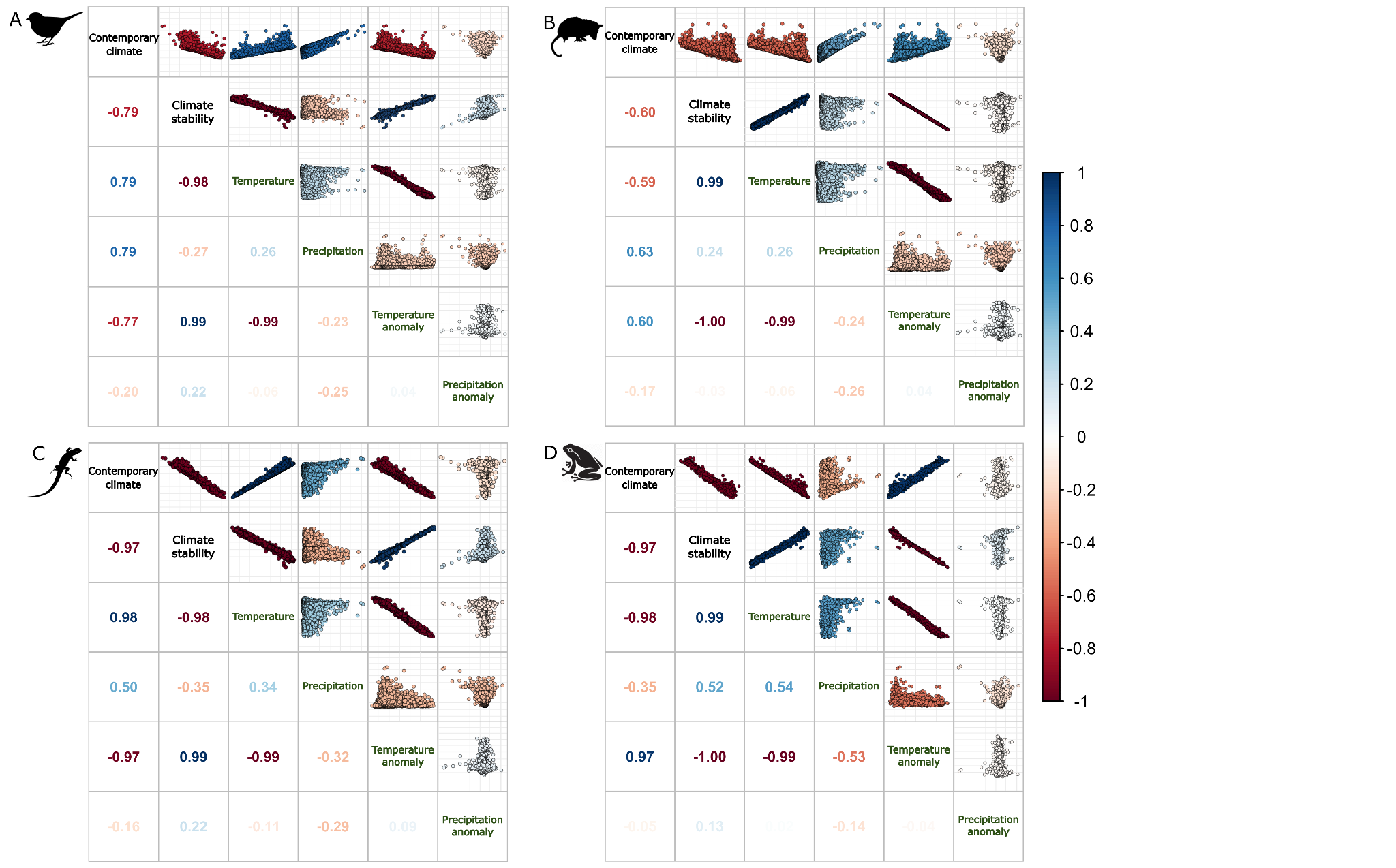


Figure S3. Correlation between the species traits variables to explain the relationship between component variables (Green) and composite variable (Black) to each taxon: (A) bird, (B) mammal, (C) squamate, and (D) amphibian.


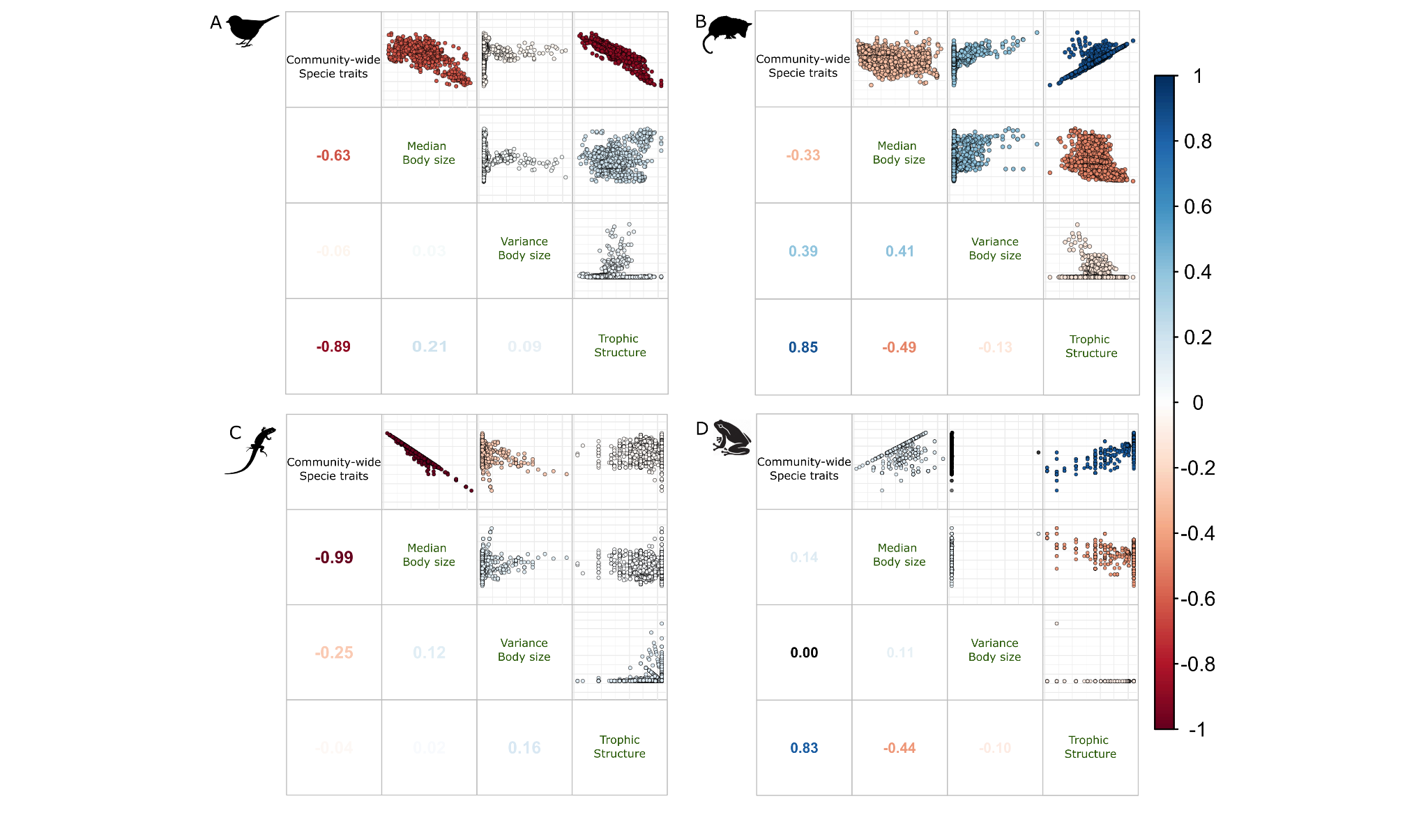


Figure S4. Latitudinal pattern of tetrapod (A) Current climate and (B) Climate instability.


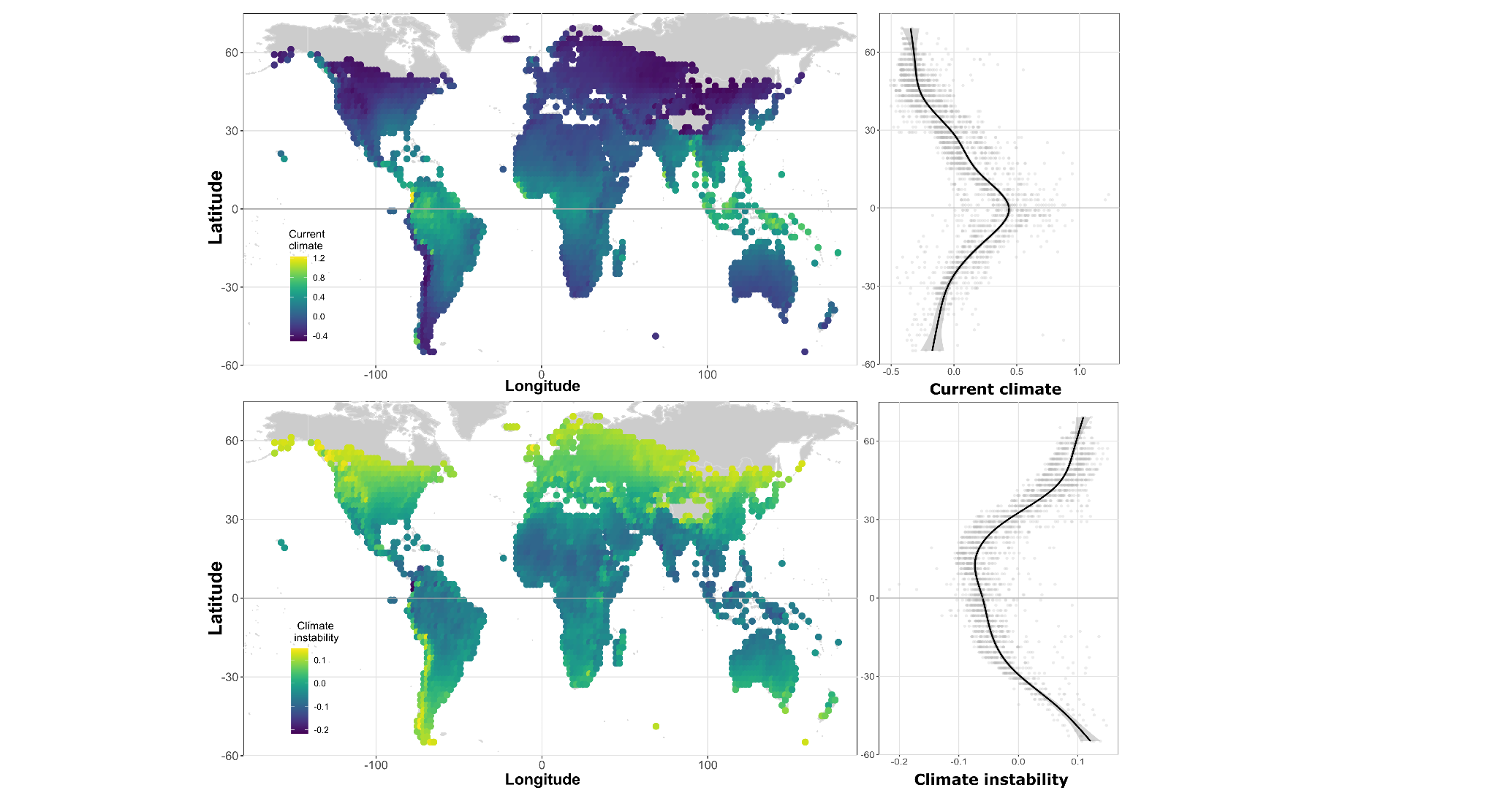


Figure S5. Latitudinal pattern of tetrapod species richness, body size,body size variance, and (D) trophic structure to (A) birds, (B) mammals, (C) squamates , and (D)amphibians.


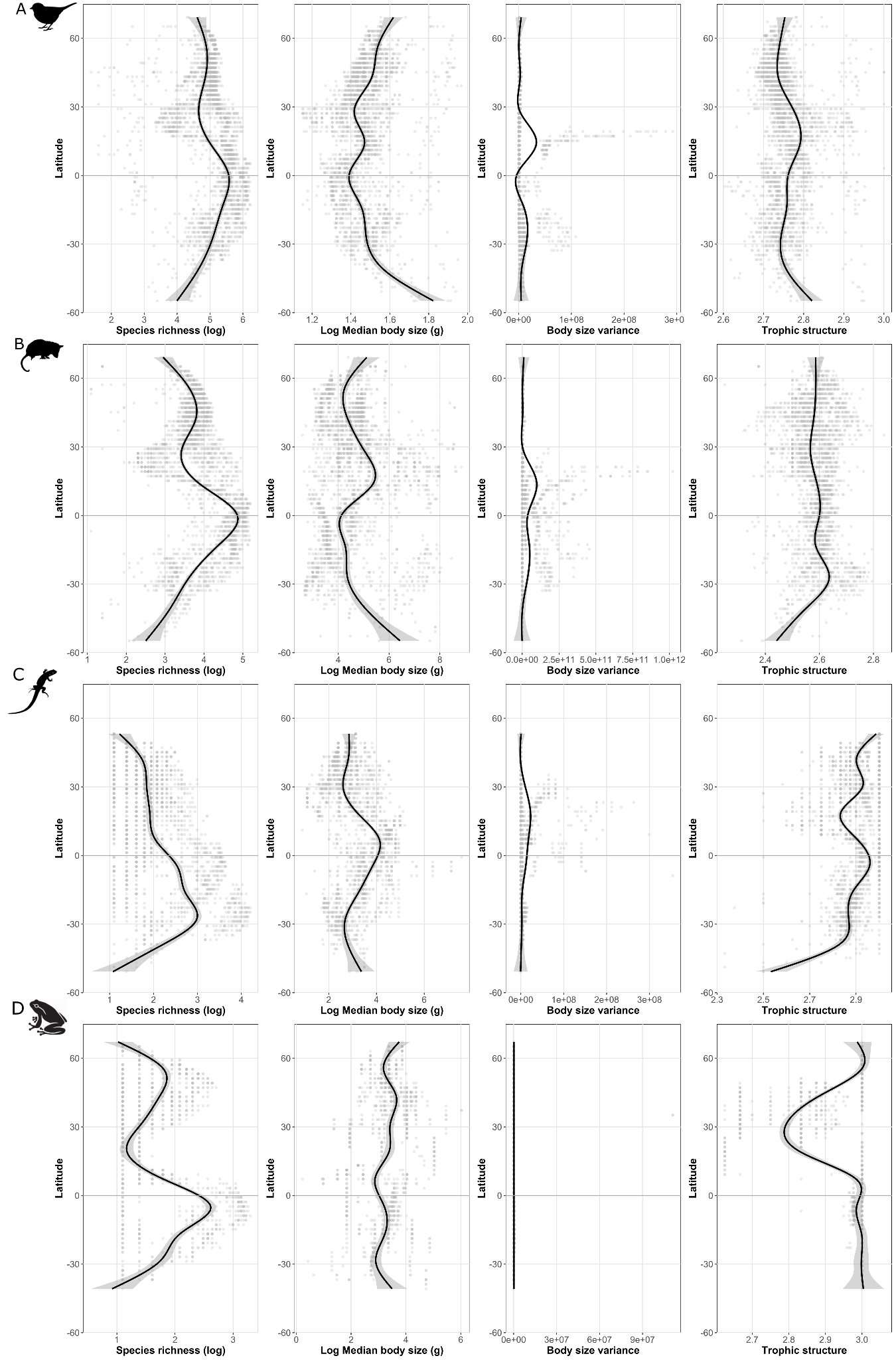


Figure S6. Density of trophic structure by group (i.e.,Birds, Mammals, Amphibians, and Squamates). Values of trophic structure next to 3 higher top-heavy structures and values next to 2 represent bottom-heavy structures.


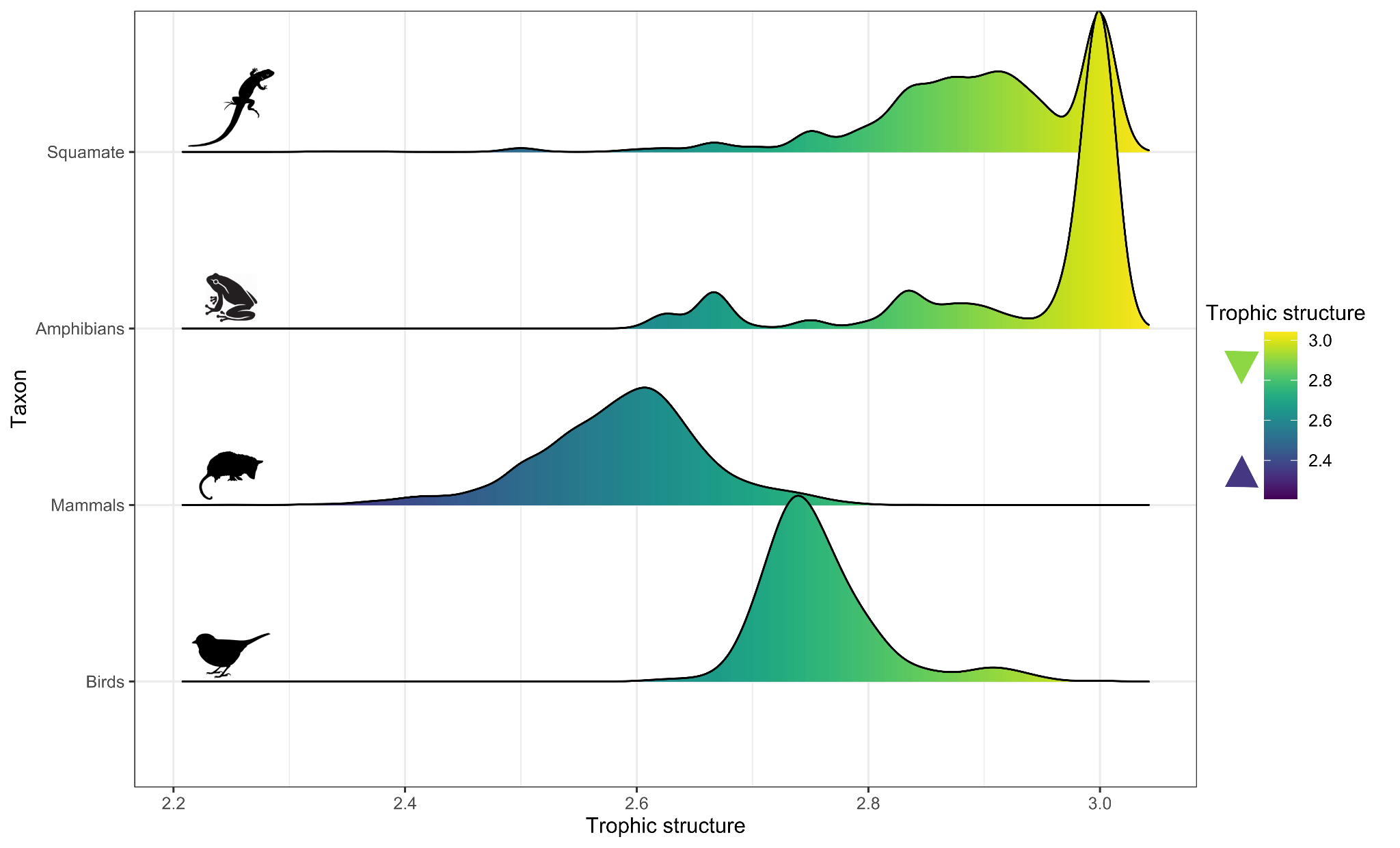


**Tables**

Table S1. Details of the structural equation of pSEM containing the composite variables and the MEMs selected (see Table S2).

| **Taxon** | **Model** | **Response Variable** | **Predictor Variable** | **Moran's I** | **Fisher’s C** | **p value** | **DF** | **R²** |
| --- | --- | --- | --- | --- | --- | --- | --- | --- |
| Bird | M_B1_ | Species Richness | Contemporary climate + Climate instability + Community-wide species traits + Selected MEMs | 0.61 | 1130 | 0.001 | 68 | 0.66 |
|  | M_B2_ | Trophic Structure | Contemporary climate + Climate instability + Selected MEMs | 0.39 |  |  |  | 0.30 |
|  | M_B3_ | Median Body size | Contemporary climate + Climate instability + Selected MEMs | 0.52 |  |  |  | 0.32 |
|  | M_B4_ | Variance Body size | Contemporary climate + Climate instability + Selected MEMs | 0.81 |  |  |  | 0.12 |
| Mammal | M_M1_ | Species Richness | Contemporary climate + Climate instability + Community-wide species traits + Selected MEMs | 0.71 | 1713 | 0.001 | 78 | 0.61 |
|  | M_M2_ | Trophic Structure | Contemporary climate + Climate instability + Selected MEMs | 0.72 |  |  |  | 0.30 |
|  | M_M3_ | Median Body size | Contemporary climate + Climate instability + Selected MEMs | 0.69 |  |  |  | 0.45 |
|  | M_M4_ | Variance Body size | Contemporary climate + Climate instability + Selected MEMs | 0.69 |  |  |  | 0.57 |
| Amphibian | M_A1_ | Species Richness | Contemporary climate + Climate instability + Community-wide species traits + Selected MEMs | 0.73 | 1101 |  |  | 0.74 |
|  | M_A2_ | Trophic Structure | Contemporary climate + Climate instability + Selected MEMs | 0.77 |  | 0.001 | 52 | 0.74 |
|  | M_A3_ | Median Body size | Composite contemporary + Climate instability + Selected MEMs | 0.63 |  |  |  | 0.34 |
|  | M_A4_ | Variance Body size | Contemporary climate + Climate instability + Selected MEMs | 0.67 |  |  |  | 0.44 |
| Squamate | M_S1_ | Species Richness | Contemporary climate + Climate instability + Community-wide species traits + Selected MEMs | 0.64 | 859 | 0.001 | 62 | 0.75 |
|  | M_S2_ | Trophic structure | Contemporary climate + Climate instability + Selected MEMs | 0.72 |  |  |  | 0.18 |
|  | M_S3_ | Median Body size | Contemporary climate + Climate instability + Selected MEMs | 0.50 |  |  |  | 0.40 |
|  | M_S4_ | Variance Body size | Contemporary climate + Climate instability + Selected MEMs | 0.33 |  |  |  | 0.33 |

Table S2. Details of relationship between variable responses and predictors obtained in the SEM models containing the composite climatic variables and the MEMs selected.

| **Taxon** | **Response** | **Predictor** | **p value** | **Std. Estimate** |
| --- | --- | --- | --- | --- |
| Mammals | **Species richness** | **Contemporary climate** | **<0.001** | **0.44** |
|  | **Species richness** | **Climate instability** | **<0.001** | **0.49** |
|  | **Species richness** | **Community-wide species trait** | **<0.001** | **0.22** |
|  | Species richness | MEM4 | <0.001 | 0.16 |
|  | Species richness | MEM8 | <0.001 | -0.26 |
|  | Species richness | MEM5 | <0.001 | 0.16 |
|  | Species richness | MEM1 | <0.001 | 0.22 |
|  | Species richness | MEM7 | <0.001 | -0.23 |
|  | Species richness | MEM2 | <0.001 | 0.13 |
|  | Species richness | MEM6 | <0.001 | -0.06 |
|  | Species richness | MEM11 | <0.001 | 0.11 |
|  | Species richness | MEM18 | <0.001 | 0.08 |
|  | Species richness | MEM22 | <0.001 | -0.09 |
|  | **Body size** | **Contemporary climate** | **<0.001** | **-0.25** |
|  | **Body size** | **Climate instability** | **<0.001** | **-0.20** |
|  | Body size | MEM4 | <0.001 | 0.33 |
|  | Body size | MEM6 | <0.001 | -0.23 |
|  | Body size | MEM1 | <0.001 | -0.24 |
|  | Body size | MEM7 | <0.001 | -0.14 |
|  | Body size | MEM10 | <0.001 | -0.16 |
|  | Body size | MEM3 | <0.001 | -0.19 |
|  | Body size | MEM8 | <0.001 | -0.09 |
|  | Body size | MEM13 | <0.001 | 0.14 |
|  | Body size | MEM11 | <0.001 | -0.13 |
|  | Body size | MEM18 | <0.001 | -0.11 |
|  | Body size | MEM2 | 0.537 | -0.01 |
|  | **Var. body size** | **Contemporary climate** | **<0.001** | **-0.08** |
|  | **Var. body size** | **Climate instability** | **<0.001** | **-0.27** |
|  | Var. body size | MEM8 | <0.001 | -0.26 |
|  | Var. body size | MEM1 | <0.001 | -0.58 |
|  | Var. body size | MEM4 | <0.001 | 0.31 |
|  | Var. body size | MEM7 | <0.001 | -0.14 |
|  | Var. body size | MEM5 | <0.001 | 0.15 |
|  | Var. body size | MEM13 | <0.001 | 0.22 |
|  | Var. body size | MEM11 | <0.001 | 0.07 |
|  | Var. body size | MEM18 | <0.001 | -0.06 |
|  | **Trophic structure** | **Contemporary climate** | **<0.001** | **0.12** |
|  | **Trophic structure** | **Climate instability** | **<0.001** | **0.31** |
|  | Trophic structure | MEM3 | <0.001 | 0.09 |
|  | Trophic structure | MEM6 | <0.001 | 0.35 |
|  | Trophic structure | MEM4 | <0.001 | -0.20 |
|  | Trophic structure | MEM7 | <0.001 | 0.11 |
|  | Trophic structure | MEM21 | <0.001 | 0.27 |
|  | Trophic structure | MEM15 | <0.001 | -0.13 |
|  | **~~ Body size** | **~~ Var. body size** | **<0.001** | **0.28** |
|  | **~~ Body size** | **~~ Trophic structure** | **<0.001** | **-0.38** |
|  | **~~ Var. body size** | **~~ Trophic structure** | **0.219** | **-0.01** |
| Bird | **Species richness** | **Contemporary climate** | **<0.001** | **0.30** |
|  | **Species richness** | **Climate instability** | **0.1655** | **-0.02** |
|  | **Species richness** | **Community-wide species trait** | **<0.001** | **0.66** |
|  | Species richness | MEM5 | <0.001 | -0.22 |
|  | Species richness | MEM4 | <0.001 | -0.13 |
|  | Species richness | MEM7 | <0.001 | 0.18 |
|  | Species richness | MEM8 | <0.001 | -0.16 |
|  | Species richness | MEM6 | <0.001 | 0.04 |
|  | Species richness | MEM26 | <0.001 | -0.09 |
|  | **Body size** | **Contemporary climate** | **<0.001** | **0.14** |
|  | **Body size** | **Climate instability** | **<0.001** | **0.29** |
|  | Body size | MEM2 | <0.001 | 0.26 |
|  | Body size | MEM6 | <0.001 | -0.32 |
|  | Body size | MEM1 | <0.001 | -0.14 |
|  | Body size | MEM8 | <0.001 | -0.08 |
|  | Body size | MEM13 | <0.001 | 0.18 |
|  | Body size | MEM3 | <0.001 | 0.11 |
|  | Body size | MEM12 | <0.001 | 0.08 |
|  | Body size | MEM9 | <0.001 | 0.11 |
|  | **Var. body size** | **Contemporary climate** | **<0.001** | **-0.58** |
|  | **Var. body size** | **Climate instability** | **<0.001** | **-0.44** |
|  | Var. body size | MEM2 | <0.001 | -0.08 |
|  | Var. body size | MEM1 | 0.76 | -0.006 |
|  | **Trophic structure** | **Contemporary climate** | **0.70** | **0.01** |
|  | **Trophic structure** | **Climate instability** | **<0.001** | **-0.12** |
|  | Trophic structure | MEM1 | <0.001 | -0.39 |
|  | Trophic structure | MEM6 | <0.001 | 0.08 |
|  | Trophic structure | MEM4 | <0.001 | -0.11 |
|  | Trophic structure | MEM7 | <0.001 | 0.05 |
|  | Trophic structure | MEM8 | <0.001 | 0.15 |
|  | Trophic structure | MEM12 | <0.001 | 0.16 |
|  | **~~ Body size** | **~~ Var. body size** | **<0.001** | **0.16** |
|  | **~~ Body size** | **~~ Trophic structure** | **<0.001** | **0.36** |
|  | **~~ Var. body size** | **~~ Trophic structure** | **<0.001** | **-0.16** |
| Amphibian | **Species richness** | **Contemporary climate** | **<0.001** | **0.77** |
|  | **Species richness** | **Climate instability** | **<0.001** | **0.92** |
|  | **Species richness** | **Community-wide species trait** | **0.300** | **-0.02** |
|  | Species richness | MEM3 | <0.001 | -0.45 |
|  | Species richness | MEM1 | <0.001 | -0.38 |
|  | Species richness | MEM2 | <0.001 | -0.33 |
|  | Species richness | MEM4 | <0.001 | -0.20 |
|  | Species richness | MEM5 | 0.132 | 0.02 |
|  | Species richness | MEM8 | 0.051 | -0.02 |
|  | Species richness | MEM6 | <0.001 | 0.06 |
|  | Species richness | MEM11 | <0.001 | -0.10 |
|  | **Body size** | **Contemporary climate** | **0.358** | **0.09** |
|  | **Body size** | **Anomaly precipitation** | **0.382** | **-0.09** |
|  | Body size | MEM5 | <0.001 | -0.30 |
|  | Body size | MEM8 | <0.001 | 0.24 |
|  | Body size | MEM1 | <0.001 | -0.16 |
|  | Body size | MEM7 | <0.001 | -0.12 |
|  | Body size | MEM2 | <0.001 | 0.09 |
|  | Body size | MEM3 | <0.001 | -0.15 |
|  | Body size | MEM12 | <0.001 | 0.19 |
|  | Body size | MEM15 | <0.001 | 0.20 |
|  | **Var. body size** | **Contemporary climate** | **<0.001** | **0.29** |
|  | **Var. body size** | **Climate instability** | **<0.001** | **0.39** |
|  | Var. body size | MEM2 | <0.001 | 0.23 |
|  | Var. body size | MEM8 | <0.001 | 0.46 |
|  | Var. body size | MEM3 | <0.001 | -0.08 |
|  | Var. body size | MEM1 | <0.001 | 0.15 |
|  | Var. body size | MEM5 | <0.001 | -0.10 |
|  | Var. body size | MEM14 | <0.001 | -0.09 |
|  | Var. body size | MEM15 | <0.001 | -0.20 |
|  | Var. body size | MEM10 | <0.001 | 0.21 |
|  | **Trophic structure** | **Contemporary climate** | **0.244** | **-0.08** |
|  | **Trophic structure** | **Climate instability** | **0.187** | **-0.10** |
|  | Trophic structure | MEM3 | <0.001 | -0.18 |
|  | Trophic structure | MEM2 | <0.001 | -0.49 |
|  | Trophic structure | MEM5 | <0.001 | 0.58 |
|  | Trophic structure | MEM4 | <0.001 | -0.17 |
|  | Trophic structure | MEM7 | <0.001 | 0.13 |
|  | Trophic structure | MEM10 | <0.001 | 0.09 |
|  | Trophic structure | MEM14 | <0.001 | 0.22 |
|  | Trophic structure | MEM6 | 0.571 | 0.01 |
|  | Trophic structure | MEM1 | <0.001 | -0.14 |
|  | **~~ Body size** | **~~ Var. body size** | **<0.001** | **0.28** |
|  | **~~ Body size** | **~~ Trophic structure** | **<0.001** | **-0.38** |
|  | **~~ Var. body size** | **~~ Trophic structure** | **<0.001** | **-0.028** |
| Squamate | **Species richness** | **Contemporary climate** | **<0.001** | **0.33** |
|  | **Species richness** | **Climate instability** | **0.337** | **0.05** |
|  | **Species richness** | **Community-wide species trait** | **<0.001** | **0.14** |
|  | Species richness | MEM3 | <0.001 | -0.23 |
|  | Species richness | MEM1 | <0.001 | -0.51 |
|  | Species richness | MEM12 | <0.001 | 0.14 |
|  | Species richness | MEM7 | <0.001 | 0.06 |
|  | Species richness | MEM5 | <0.001 | -0.40 |
|  | Species richness | MEM8 | <0.001 | 0.14 |
|  | Species richness | MEM6 | <0.001 | 0.18 |
|  | Species richness | MEM11 | 0.667 | -0.006 |
|  | Species richness | MEM18 | <0.001 | 0.12 |
|  | **Body size** | **Contemporary climate** | **<0.001** | **0.91** |
|  | **Body size** | **Climate instability** | **<0.001** | **0.76** |
|  | Body size | MEM4 | <0.001 | 0.30 |
|  | Body size | MEM6 | <0.001 | -0.17 |
|  | Body size | MEM1 | <0.001 | 0.24 |
|  | Body size | MEM5 | <0.001 | 0.08 |
|  | Body size | MEM2 | <0.001 | -0.26 |
|  | Body size | MEM3 | <0.001 | 0.09 |
|  | Body size | MEM9 | <0.001 | -0.14 |
|  | **Var. body size** | **Contemporary climate** | **<0.001** | **1.13** |
|  | **Var. body size** | **Climate instability** | **<0.001** | **0.55** |
|  | Var. body size | MEM2 | <0.001 | 0.24 |
|  | Var. body size | MEM6 | <0.001 | 0.16 |
|  | Var. body size | MEM13 | <0.001 | 0.13 |
|  | Var. body size | MEM9 | <0.001 | -0.09 |
|  | Var. body size | MEM1 | <0.001 | -0.22 |
|  | Var. body size | MEM5 | <0.001 | -0.16 |
|  | Var. body size | MEM4 | <0.001 | -0.05 |
|  | Var. body size | MEM11 | <0.001 | -0.12 |
|  | Var. body size | MEM10 | <0.001 | 0.08 |
|  | **Trophic structure** | **Contemporary climate** | **<0.001** | **0.37** |
|  | **Trophic structure** | **Climate instability** | **<0.001** | **0.29** |
|  | Trophic structure | MEM3 | <0.001 | -0.14 |
|  | Trophic structure | MEM2 | <0.001 | 0.16 |
|  | Trophic structure | MEM9 | <0.001 | 0.14 |
|  | Trophic structure | MEM4 | <0.001 | 0.21 |
|  | Trophic structure | MEM7 | <0.001 | 0.12 |
|  | Trophic structure | MEM10 | <0.001 | -0.09 |
|  | Trophic structure | MEM13 | <0.001 | 0.13 |
|  | Trophic structure | MEM1 | 0.031 | 0.05 |
|  | **~~ Body size** | **~~ Var. body size** | **<0.001** | **0.27** |
|  | **~~ Body size** | **~~ Trophic structure** | **0.003** | **-0.07** |
|  | **~~ Var. body size** | **~~ Trophic structure** | **<0.001** | **-0.14** |

Table S3. Details of evaluated ordinary least squares (OLS) models with strong correlated variables. Values of correlated variables could be observed on Figure S2. Selected models with lower Akaike Information Criterion (AIC) are in bold.

| **Group** | **Correlated variables on Models** | **AIC** | **p value** |
| --- | --- | --- | --- |
| Bird | Temperature and Anomaly of temperature | **690.04** | 0.365 |
|  | Only with temperature | 690.25 |  |
| Mammals | Temperature and Anomaly of temperature | **772.09** | <0.001 |
|  | Only with temperature | 779.77 |  |
| Amphibians | Temperature and Anomaly of temperature | **261.65** | <0.001 |
|  | Only with temperature | 288.25 |  |
| Squamates | Temperature and Anomaly of temperature | **905.25** | <0.001 |
|  | Only with temperature | 956.10 |  |
